# Dynamic Dissection of the Endocytosis of Porcine Epidemic Diarrhea Coronavirus Cooperatively Mediated by Clathrin and Caveolae as Visualized by Single-Virus Tracking

**DOI:** 10.1128/mBio.00256-21

**Published:** 2021-03-30

**Authors:** Yangyang Li, Jian Wang, Wei Hou, Yanke Shan, Shouyu Wang, Fei Liu

**Affiliations:** a Joint International Research Laboratory of Animal Health and Food Safety of Ministry of Education and Single Molecule Nanometry Laboratory (Sinmolab), Nanjing Agricultural University, Nanjing, Jiangsu, China; b Computational Optics Laboratory, Jiangnan University, Wuxi, Jiangsu, China; Columbia University Medical College

**Keywords:** porcine epidemic diarrhea coronavirus, single-virus tracking, endocytic pathway, endosome trafficking, viral fusion

## Abstract

Coronaviruses (CoVs) have caused severe diseases in humans and animals. Endocytic pathways, such as clathrin-mediated endocytosis (CME) and caveolae-mediated endocytosis (CavME), play an important role for CoVs to penetrate the cell membrane barrier. In this study, a novel CoV entry manner is unraveled in which clathrin and caveolae can cooperatively mediate endocytosis of porcine epidemic diarrhea coronavirus (PEDV). Using multicolor live-cell imaging, the dynamics of the fluorescently labeled clathrin structures, caveolae structures, and PEDV were dissected. During CavME of PEDV, we found that clathrin structures can fuse with caveolae near the cell plasma membrane, and the average time of PEDV penetrating the cell membrane was within ∼3 min, exhibiting a rapid course of PEDV entry. Moreover, based on the dynamic recruitment of clathrin and caveolae structures and viral motility, the direct evidence also shows that about 20% of PEDVs can undergo an abortive entry via CME and CavME. Additionally, the dynamic trafficking of PEDV from clathrin and caveolae structures to early endosomes, and from early endosomes to late endosomes, and viral fusion were directly dissected, and PEDV fusion mainly occurred in late endosomes within ∼6.8 min after the transport of PEDV to late endosomes. Collectively, this work systematically unravels the early steps of PEDV infection, which expands our understanding of the mechanism of CoV infection.

## Abstract (precis)

Emerging and re-emerging coronaviruses cause serious human and animal epidemics worldwide. For many enveloped viruses, including coronavirus, it is evident that breaking the plasma membrane barrier is a pivotal and complex process, which contains multiple dynamic steps.

## INTRODUCTION

Coronaviruses (CoVs) are a group of enveloped viruses with nonsegmented, single-stranded, and positive-sense RNA viruses (1, 2). The current ongoing outbreak of coronavirus disease 2019 (COVID-19) is caused by severe acute respiratory syndrome coronavirus 2 (SARS-CoV-2) and is a serious threat to human health (3–5). Additionally, coronavirus, such as porcine epidemic diarrhea coronavirus (PEDV), can also cause severe disease in animals, resulting in huge economic losses in the swine industry (6, 7). However, we still lack an effective CoV antiviral therapeutic strategy. The hijacking of host endocytic trafficking is an important way for CoVs to penetrate the cell membrane barrier. Unraveling the endocytic pathways of CoVs may therefore provide a potential therapeutic strategy for treating CoV infection.

PEDV is a major causative agent of swine enteric disease in pigs of all ages. It was initially reported in the United Kingdom and Belgium in the early 1970s, and a highly pathogenic variant strain was identified in 2010, which caused high morbidity of up to 100% in piglets. The threat of PEDV has been constant since then (8–10). With the lack of a basic understanding of PEDV infection and the emergence of highly pathogenic strains of PEDV, current antiviral therapeutic strategies cannot provide complete protection for PEDV infection. To obtain detailed insights into the mechanism of PEDV, a systematic and comprehensive dissection of PEDV entry is urgently needed.

The PEDV genome is about 28 kb long and contains a 5′ untranslated region (UTR), at least seven open reading frames (ORFs), and a 3′ UTR (11). Among the viral ORFs, ORF 2 encodes the PEDV spike (S) glycoprotein, which is assembled into trimers on the surface of the virion to mediate PEDV entry. Similar to other coronavirus (CoV) S glycoproteins, the PEDV S glycoprotein possesses two functional subunits as follows: the S1 subunit is responsible for binding to cell surface receptors and the S2 subunit is responsible for mediating viral fusion. Receptor binding and acidic pH can initiate conformational changes of the PEDV S glycoprotein, driving fusion of the viral envelope membrane with the cell membrane to release the viral genome into the cytoplasm (12). Although great efforts have been made to understand the mechanisms of PEDV infection, such as viral genome structures, S glycoprotein conformational changes, and receptor binding in the early stages of PEDV infection, many crucial questions regarding the endocytic pathway, endosome trafficking, and viral fusion remain incompletely understood.

First, the endocytic pathway of PEDV remains controversial. Previous studies indicate that PEDV entry into cells is via clathrin-mediated endocytosis (CME) and independent of caveolae-mediated endocytosis (CavME) (13). But the later study illustrated that methyl-β-cyclodextrin, a CavME inhibitor, had a significant inhibitory effect on PEDV infection during the pre-entry period, indicating that PEDV may also use CavME to enter cells (14). Moreover, a very recent study indicated that PEDVs can enter into cells via both CME and CavME (15). CME involves internalization of ligand-receptor cargos via clathrin-coated pits (CCPs), which is the main endocytic pathway in mammalian cells (16, 17). CavME is a clathrin-independent endocytic pathway, which involves 50- to 100-nm bulb-shaped invaginations called caveolae (18, 19). Without direct real-time imaging of PEDV infection in living cells, it is still unclear whether CCPs or caveolae uptake PEDV particles or whether this is the real scenario of PEDV infection in CCP and caveolae double-labeled cells, which is vital for understanding the role of CCPs and caveolae in PEDV entry.

Next, the identity of the endosomes in which PEDV fusion occurs also remains elusive. A very recent study on PEDV infection demonstrates that PEDV particles can colocate with the early and late endosomes/lysosome marks, and viral infectivity was dramatically reduced with inhibition of endosomal acidification (15). This finding indicates that PEDV may mediate membrane fusion within acidic endosomes. However, static images and ensemble cell assays cannot exhibit the dynamic fate of PEDV particles, which involves the interaction of cellular organelles and viral fusion in living cells. Single-virus tracking can provide direct evidence of the exact manner of viral infection by dynamically observing individual viral particles in living cells with high spatiotemporal resolution (20, 21). Thus, it is an ideal tool to answer the crucial questions outlined above.

Here, we imaged the dynamics of the fluorescently labeled clathrin, caveolae, and PEDV in living cells at the single-particle level. To make a productive infection, CME and CavME of PEDV were confirmed by directly observing the *de novo* formation of CCPs and caveolae at the viral binding sites, and a novel CoVs entry manner was also uncovered in which clathrin and caveolae cooperatively mediated endocytosis (C^3^ME) of PEDV. Moreover, based on the dynamic recruitment of clathrin and caveolae structures and viral motility, about a proportion of 20% PEDV undergoing an abortive endocytosis was discovered. After PEDV enters into endosomes, PEDV fusion mainly occurred in late endosomes. These results provide detailed evidence that PEDV hijacks multiple endocytic pathways, including CME, CavME, and C^3^ME, to make a productive infection and hijacks existing abortive endocytosis of PEDV and PEDV fusion. This work directly demonstrates the cooperation of CME and CavME for viral productive infection, such as C^3^ME, which expands our understanding of the early stages of viral infection.

## RESULTS

### Characterization of DiD-labeled PEDVs and fluorescent protein-labeled endocytic structures.

**FIG 1:**
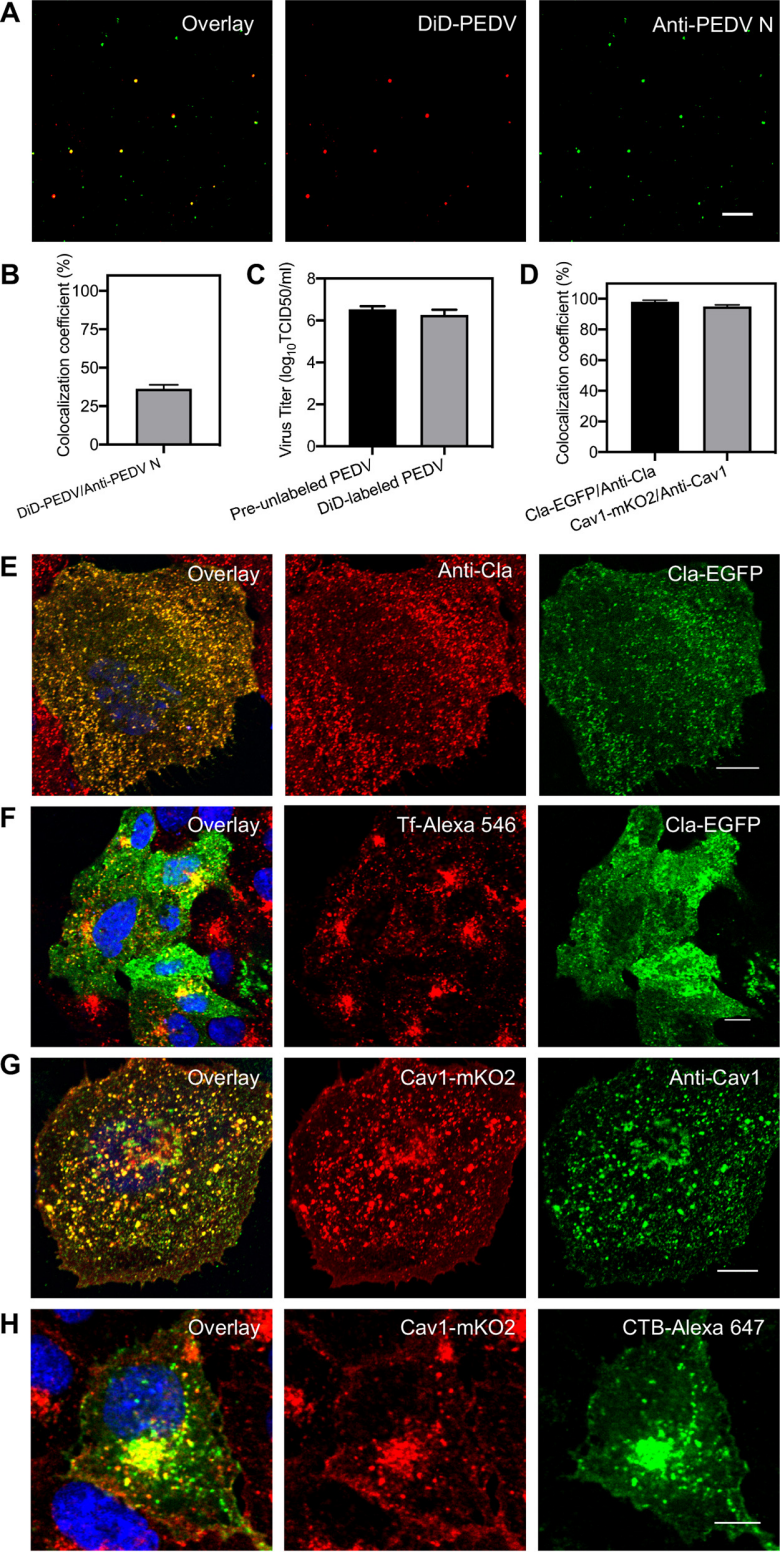
Characterization of DiD-labeled PEDVs and fluorescent protein-labeled endocytic structures. (A) Fluorescence images of DiD-labeled and immunostained PEDVs. (B) The efficiency of viral labeling by DiD dyes evaluated by the colocalization of DiD signal and anti-PEDV N protein signal. (C) Infectivity assay of preunlabeled PEDVs and DiD-labeled PEDVs measured by TCID_50_; the average of logarithms of TCID_50_ values in 3 independent experiments of preunlabeled PEDVs was 6.53 and that of DiD-labeled PEDVs was 6.26. (D) Colocalization analysis of discrete fluorescent protein signal (Cla-EGFP) and immunofluorescence signal (anti-Cla) of clathrin structures and fluorescent protein signal (Cav1-mKO2) and immunofluorescence signal (anti-Cav1) of caveolae structures, respectively. (E) Representative immunofluorescence images of clathrin-coated structures in Vero CCL81 cells. (F) Uptake of Tf in Vero-CCL81 cells expressing Cla-EGFP. (G) Representative immunofluorescence images of caveolae structures in Vero-CCL81. (H) Uptake of CTB subunit in Vero-CCL81 cells expressing Cav1-mKO2. In all panels, data shown are mean results ± standard deviation (SD) of three independent experiments, and white bars indicate 10 μm.

A lipophilic 1,1′-dioctadecyl-3,3,3′,3′-tetramethylindodicarbocyanine (DiD) dye which can be incorporated into the viral envelope was used to label PEDVs, and the characteristics of DiD-labeled PEDVs were tested using an immunofluorescence assay. In the presence of Polybrene in viral supernatants, DiD-labeled PEDVs were immobilized on coverslips and then incubated with anti-PEDV N primary antibody and fluorescein isothiocyanate (FITC)-conjugated secondary antibody for PEDV N protein labeling (Fig. 1A). Analysis indicates that about 39% FITC-labeled particles exhibited DiD fluorescence signals (Fig. 1B), meaning the efficiency of DiD dye labeling is similar to that of other enveloped viruses. Meanwhile, viral titers of equal amounts of previously DiD-unincubated PEDVs (preunlabeled PEDVs) and the harvested DiD-incubated PEDVs (DiD-labeled PEDVs) were determined by 50% tissue culture infective dose (TCID_50_) (Fig. 1C). The average of logarithms of TCID_50_ values in three independent experiments of preunlabeled PEDVs was 6.53 and that of DiD-labeled PEDVs was 6.26, illustrating that the viral titer reduction of DiD-labeled PEDVs was only 4% compared with that of preunlabeled PEDVs. Therefore, PEDVs were labeled with lipophilic DiD dyes with satisfactorily efficient fluorescence signals and low influence on viral infectivity.

A Cla-enhanced green fluorescent protein (EGFP) plasmid was constructed to label clathrin-coated structures, and the characteristics of Cla-EGFP plasmid-labeled clathrin-coated structures were tested using an immunofluorescence assay. Over 98% of discrete structures in the immunofluorescence image were colocalized with those in the EGFP image (Fig. 1D and E), illustrating that the functional integrity of clathrin was not compromised by the Cla-EGFP plasmid. Moreover, transferrin (Tf), a classic CME marker, accumulated around the nuclei, and over 95% of Tf-546 was colocalized with the clathrin-coated structures in cells expressing the Cla-EGFP plasmid (Fig. 1F), revealing that Cla-EGFP plasmid expression did not influence transferrin internalization. Both results support the use of the Cla-EGFP plasmid for clathrin-coated structure labeling.

Similarly, a Cav1-mKO2 plasmid was constructed to label caveolae endocytic structures, and the characteristics of Cav1-mKO2 plasmid-labeled caveolae endocytic structures were tested using an immunofluorescence assay. Over 95% of discrete structures in the immunofluorescence image were colocalized with those in the mKO2 image (Fig. 1D and G), illustrating that the functional integrity of caveolae was not compromised by the Cav1-mKO2 plasmid. Moreover, cholera toxin B subunit (CTB), a commonly used CavME marker, accumulated around the nuclei and over 95% of CTB-Alexa 647 was colocalized with caveolae endocytic structures in cells expressing the Cav1-mKO2 plasmid (Fig. 1H), revealing that Cav1-mKO2 plasmid expression did not influence CTB internalization. These results support the use of the Cav1-mKO2 plasmid for caveolae endocytic structure labeling.

### Productive clathrin-mediated endocytosis of PEDV.

**FIG 2:**
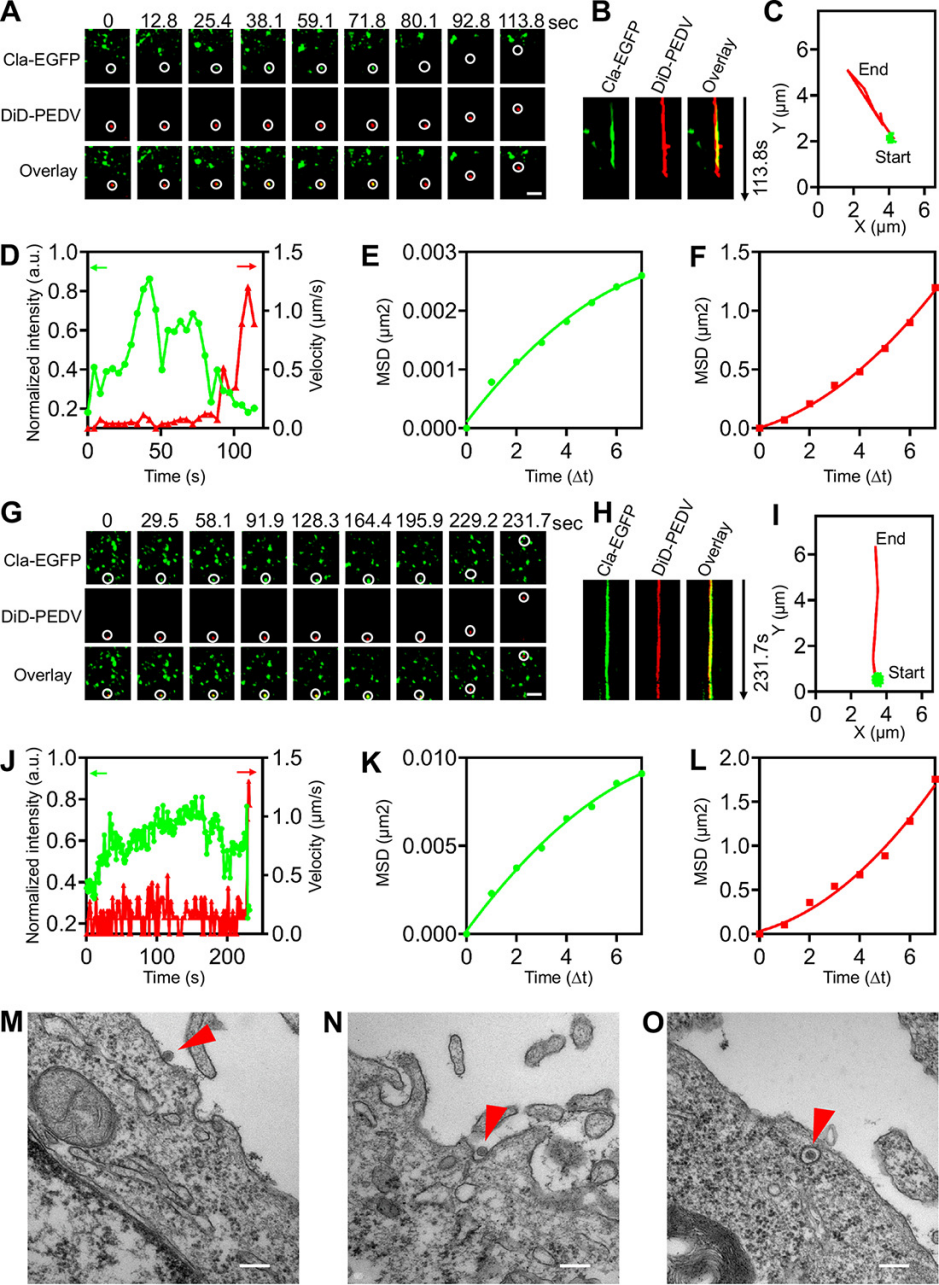
Productive CME of PEDV. (A) Time-lapse images of PEDV (red) entering into cells expressing Cla-EGFP (green). Scale bar, 2 μm. (B) Kymographs at viral binding sites. (C) Trajectories of PEDV infection during viral binding (green) and moving (red) stages. (D) Time-lapse Cla-EGFP fluorescence intensities at viral binding sites and PEDV velocities. (E, F) MSD plots of the PEDV movements during viral binding and moving stages, respectively. (G to L) Another set of time-lapse images and analysis of PEDV entry via CME. Scale bar, 2 μm. (M to O) Ultrastructural analysis on CME of PEDV, and in O, PEDV indicated with arrow was invaginated into a fuzzy “bristle-like” coated vesicle. Scale bar, 200 nm.

CME is a common route for viral internalization, and single-virus tracking was adopted to observe the dynamics of PEDV internalization via CME (Fig. 2; see Movie S1a and b in the supplemental material). In two representative time-lapse images of the CME of PEDVs (Fig. 2A and G), the clathrin protein gradually accumulated around the viruses, as *de novo* clathrin-coated pit (CCP) formation was directly observed, which is also revealed by the colocalization between DiD-labeled PEDVs and *de novo*-formed CCPs shown in kymographs of time-lapse images at viral binding sites (Fig. 2B and H). Moreover, the trajectories (Fig. 2C and I) show there were two stages of PEDV internalization. First, in the viral binding stage, PEDVs recruited the *de novo* formation of CCPs, revealed by the increased Cla-EGFP fluorescence intensity, and exhibited a rather slow speed (Fig. 2D and J) with restricted movement (Fig. 2E and K). Second, in the viral moving stage, PEDVs entered into cells shortly after the Cla-EGFP fluorescence signal disappeared, with an accelerated speed (Fig. 2D and J) and directed movement (Fig. 2F and L). Both of these examples illustrate that PEDVs can enter cells via CME.

10.1128/mBio.00256-21.3 MOVIE S1 (a, b) Productive endocytosis of PEDV (red) via CME in Vero CCL81 cells expressing Cla-EGFP (green), corresponding to the time-lapse images in Fig. 2. (c, d) PEDVs entered into cells without the formation of CCPs, corresponding to the time-lapse images in Fig. S1. In the movie, all the frames were processed with deconvolution and denoising with a Gaussian spatial filtering. The movies were generated with the NIS-Elements AR 4.51.00 (Nikon, Japan) software. The size of the movie is the same with the corresponding time-lapse images in the manuscript. Download Movie S1, MOV file, 0.6 MB. Copyright © 2021 Li et al. 2021 Li et al. https://creativecommons.org/licenses/by/4.0/ This content is distributed under the terms of the Creative Commons Attribution 4.0 International license.

10.1128/mBio.00256-21.1 FIG S1 PEDVs entered into cells without the formation of CCPs. (A, G) Time-lapse images of two individual PEDVs (red) entered into cells expressing Cla-EGFP (green) without the formation of CCPs. Scale bar, 2 μm. (B, H) Kymographs at viral binding sites. (C, I) Trajectories of PEDV infection during viral binding (green) and moving (red) stages. (D, J) Time-lapse Cla-EGFP fluorescence intensities at viral binding sites and PEDV velocities.(E, F, K, L) MSD plots of the PEDV movements during viral binding and moving stages, respectively. Download FIG S1, TIF file, 0.6 MB. Copyright © 2021 Li et al. 2021 Li et al. https://creativecommons.org/licenses/by/4.0/ This content is distributed under the terms of the Creative Commons Attribution 4.0 International license.

To investigate the CME of PEDV at an ultrastructural level, transmission electron microscopy (TEM) was employed to observe the details of PEDV internalization (Fig. 2M to O). A PEDV was first attached to the cell surface (Fig. 2M) and then induced a fuzzy “bristle-like” coated invagination (Fig. 2N); finally, an invagination was transformed into a clathrin-coated vesicle completely compassing a PEDV (Fig. 2O).

**FIG 3:**
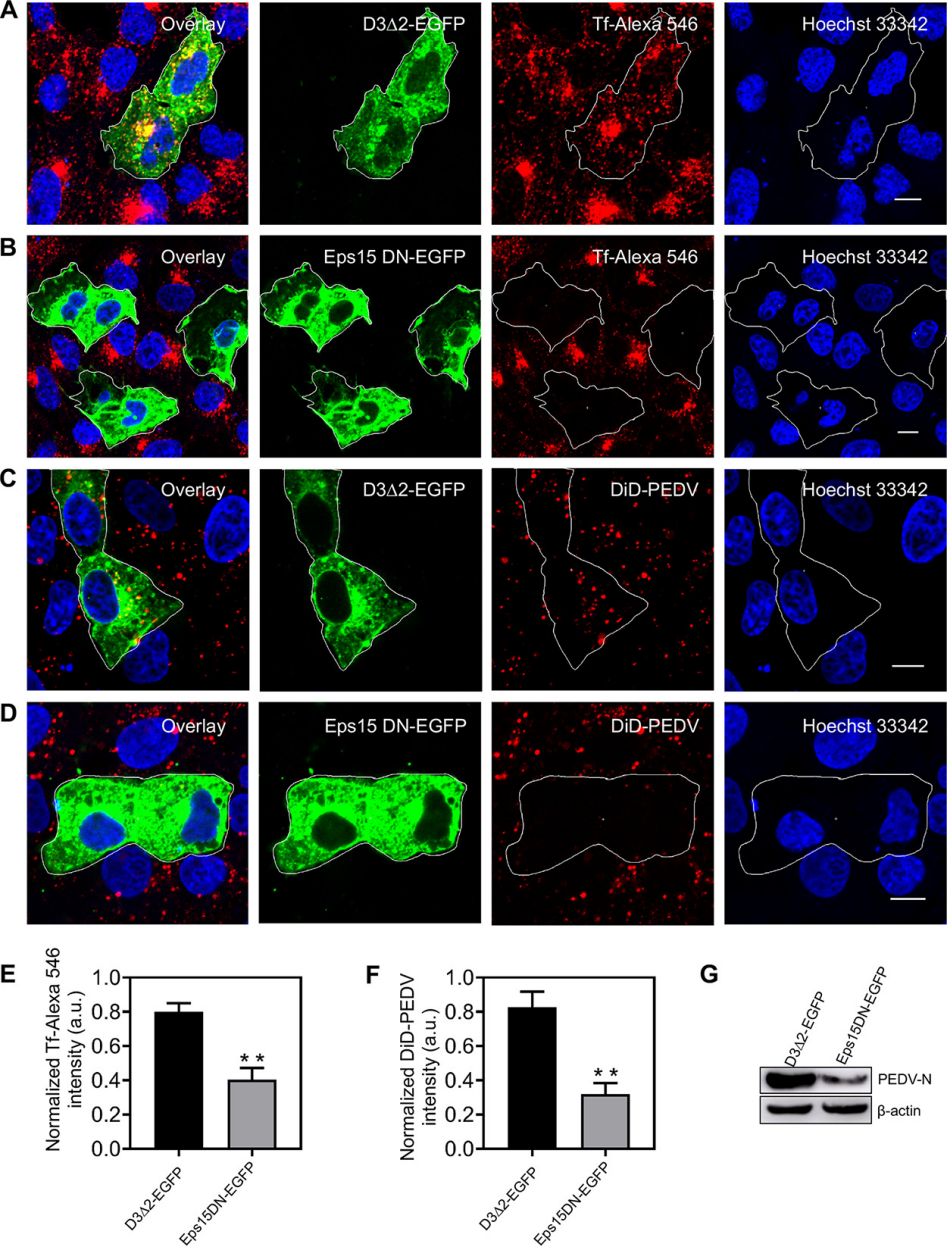
Disruption of CME by overexpressing Eps15 DN hampers PEDV entry. (A, B) Fluorescence images of the uptake of transferrin in cells expressing D3Δ2-EGFP and Eps15 DN-EGFP. As a verification of Eps15 DN inhibition efficiency, Vero CCL81 cells were transfected with plasmid D3Δ2-EGFP or Eps15 DN-EGFP; after 24 h of transfection, cells were incubated with Tf-Alexa 546 at 37°C for 30 min and then fixed and imaged. (C, D) Fluorescence images of the uptake of DiD-labeled PEDV in cells expressing D3Δ2-EGFP or Eps15 DN-EGFP. Vero CCL81 cells were transfected with plasmid D3Δ2-EGFP or Eps15 DN-EGFP; after 24 h of transfection, cells were infected with DiD-labeled PEDVs (MOI, 10) at 37°C for 30 min and then fixed and imaged. (E, F) To quantify the internalization dependency of Tf and PEDVs on the expression of each plasmid, in cells transfected with plasmid D3Δ2-EGFP or Eps15 DN-EGFP, fluorescence intensities of Tf-Alexa 546 and DiD-labeled PEDV were measured and normalized according to those of untransfected cells. Error bars denote mean ± standard deviation of three independent experiments. (G) Cells were transfected with plasmid D3Δ2-EGFP or Eps15 DN-EGFP. After a 24-h transfection, cells were infected with PEDVs (MOI, 1) and cultured at 37°C for 6 h. Then, samples were collected and analyzed by Western blotting with corresponding antibodies. PEDV-infected cells transfecting the D3Δ2-EGFP plasmid served as controls. **, *P* < 0.01. Scale bar, 10 μm.

To further confirm CME of PEDV, two plasmids, namely, Eps15DN and D3Δ2(WT), were used in interference experiments. Eps15DN, a dominant-negative mutant of Eps15, specifically interferes with the formation of CCPs at the plasma membrane. D3Δ2(WT), another form of Eps15, has no effect on CME. Compared with D3Δ2 expression, Eps15DN expression not only remarkably reduced the transferrin internalization but also significantly decreased PEDV internalization (Fig. 3), proving that CME is involved in PEDV internalization.

Taken together, results obtained by single-virus tracking as well as TEM and dominant-negative mutant interference experiments prove that CME is a productive pathway for PEDV internalization. However, instances of PEDV entering cells without CCPs were also observed by single-virus tracking in living cells (see Fig. S1 in the supplemental material; Movie S1c and d). The viral binding and moving stages of PEDVs in Fig. S1 have similar velocities, trajectories, and movements to those shown in Fig. 2. Therefore, we speculate that PEDV may enter cells via CavME.

### Productive caveolae-mediated endocytosis of PEDV.

**FIG 4:**
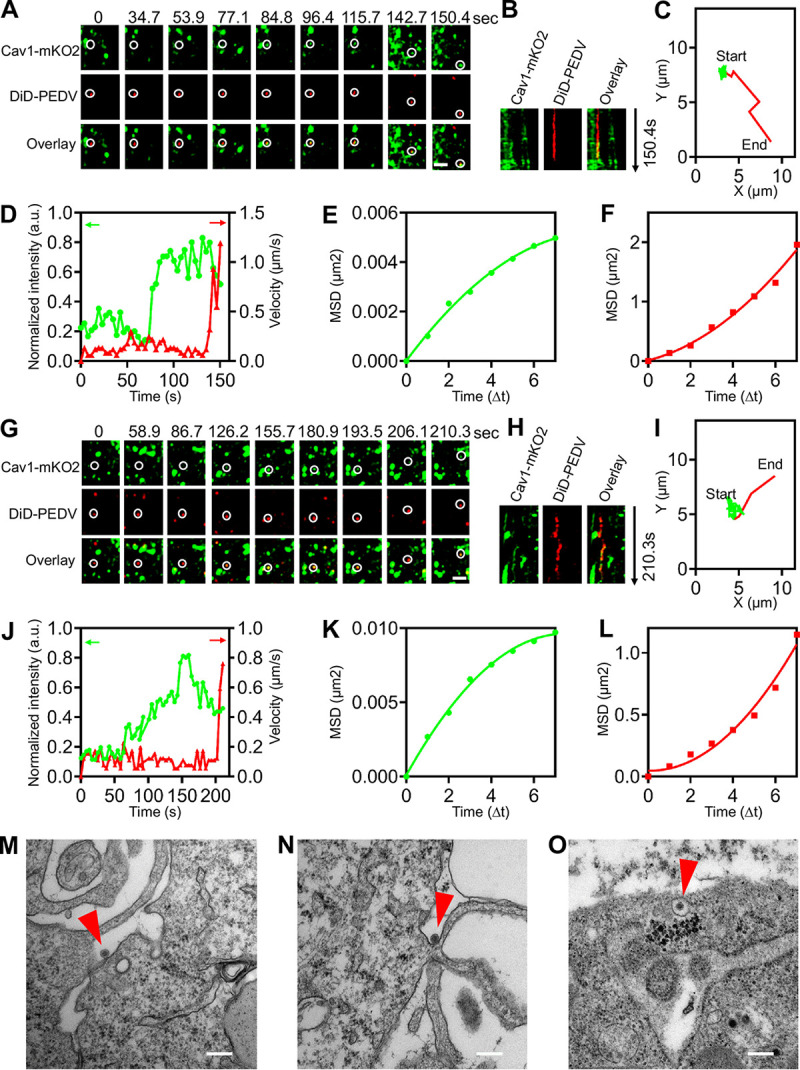
Productive CavME of PEDV. (A) Time-lapse images of two PEDVs (red) entering cells expressing Cav1-mKO2 (green). Scale bar, 2 μm. (B) Kymographs at viral binding site. (C) Trajectories of PEDV infection during viral binding (green) and moving (red) stages. (D) Time-lapse Cla-EGFP fluorescence intensities at viral binding sites and PEDV velocities. (E, F) MSD plots of the PEDV movements during viral binding and moving stages, respectively. (G to L) Another set of time-lapse images and analysis of PEDV entry via CME. Scale bar, 2 μm. (M to O) Ultrastructural analysis on CavME of PEDV, and in O, PEDV indicated with arrow was invaginated in a smooth vesicle. Scale bar, 200 nm.

In addition to CME, we found that PEDVs also entered cells via CavME. Here, single-virus tracking was also used to observe the dynamics of PEDV internalization via CavME (Fig. 4; see Movie S2a and b in the supplemental material). In two representative time-lapse images of the CavME of PEDVs (Fig. 4A and G), caveolin-1 protein accumulation around viruses was directly observed as *de novo* caveolae formation. This finding was further supported by the colocalization between DiD-labeled PEDVs and *de novo*-formed caveolae visible in kymographs of time-lapse images at the viral binding site (Fig. 4B and H). Moreover, the trajectories (Fig. 4C and I) showed that there were still two stages of PEDV internalization. First, in the viral binding stage, PEDVs recruited caveolae, revealed by the increased Cav1-mKO2 fluorescence intensity, and exhibited rather slow speed (Fig. 4D and J) and restricted movement (Fig. 4E and K). Second, in the viral moving stage, PEDVs entered cells rapidly (Fig. 4D and J) with directed movement (Fig. 4F and L). Unlike CME of PEDV, the Cav1-mKO2 fluorescence intensity did not decrease when PEDVs entered cells via CavME. These examples illustrate that PEDVs can enter cells via CavME.

10.1128/mBio.00256-21.4 MOVIE S2 (a, b) Productive endocytosis of PEDV (red) via CavME in Vero CCL81 cells expressing Cav1-mKO2 (green), corresponding to the time-lapse images in Fig. 4. Download Movie S2, MOV file, 0.4 MB. Copyright © 2021 Li et al. 2021 Li et al. https://creativecommons.org/licenses/by/4.0/ This content is distributed under the terms of the Creative Commons Attribution 4.0 International license.

To investigate CavME of PEDV at an ultrastructural level, TEM was used to show that PEDVs can enter cells via smooth noncoated invagination (Fig. 4M to O). PEDV was first attached at the cell surface (Fig. 4M) and then induced a noncoated invagination (Fig. 4N); an invagination was also transformed into a caveolae vesicle, completely compassing a PEDV (Fig. 4O).

**FIG 5:**
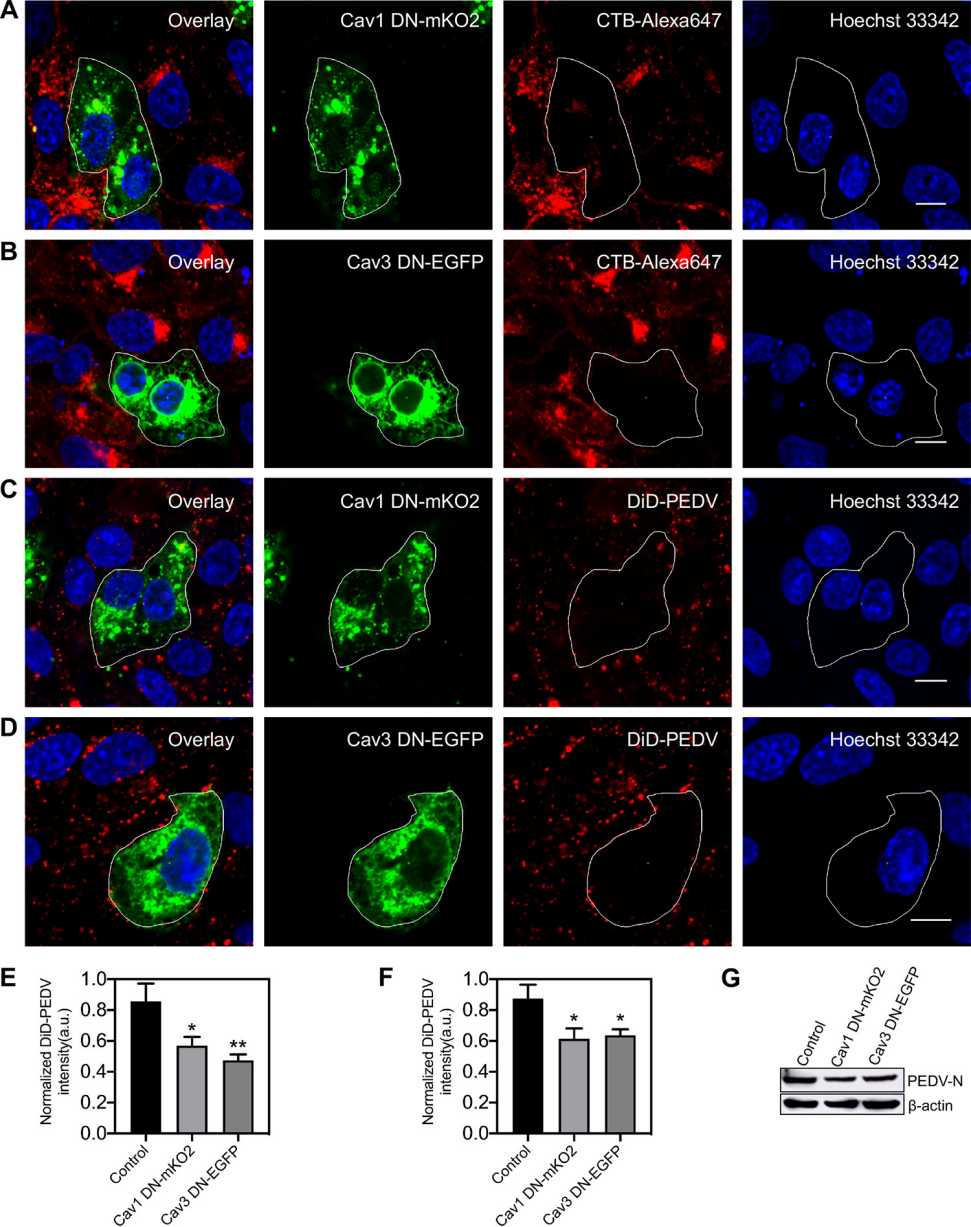
Disruption of CavME by overexpressing Cav1 DN or Cav3 DN hampers PEDV entry. (A, B) Fluorescence images of the uptake of CTB in cells expressing Cav1 DN-mKO2 or Cav3 DN-EGFP. As a verification of the dominant-negative mutant inhibition efficiency, Vero CCL81 cells were transfected with plasmid Cav1 DN-mKO2 or Cav3 DN-EGFP; after a 24-h transfection, cells were incubated with CTB-Alexa 647 for 30 min and then fixed and imaged. (C, D) Fluorescence images of the uptake of DiD-labeled PEDVs in cells expressing Cav1 DN-mKO2 or Cav3 DN-EGFP. Vero CCL81 cells were transfected with plasmid Cav1 DN-mKO2 or Cav3 DN-EGFP; after a 24-h transfection, cells were infected with DiD-labeled PEDVs (MOI, 10) for 30 min and then fixed and imaged. (E, F) To quantify the internalization dependency of CTB and PEDVs on expression of each plasmid, in cells transfected with plasmid Cav1 DN-mKO2 or Cav3 DN-EGFP, fluorescence intensities of CTB-Alexa 647 and DiD-labeled PEDV were measured and normalized according to those of untransfected cells. Error bars denote mean ± standard deviation of three independent experiments. (G) Cells were transfected with plasmid Cav1 DN-mKO2 or Cav3 DN-EGFP. After 24 h of transfection, cells were infected with PEDVs (MOI, 1) and cultured at 37°C for 6 h. Then, samples were collected and analyzed by Western blotting with corresponding antibodies. PEDV-infected untransfected cells served as controls. *, *P* < 0.05; **, *P* < 0.01. Scale bar, 10 μm.

To further confirm CavME of PEDV, two dominant-negative plasmids, namely, Cav1DN and Cav3DN, that both specifically interfere with caveolae at the plasma membrane were used in interference experiments. Compared with nontransfected cells, Cav1DN and Cav3DN both significantly reduced CTB and PEDV internalization (Fig. 5), proving that CavME is involved in PEDV internalization. The results of single-virus tracking as well as from TEM and interference experiments illustrate that in addition to CME, CavME is also a productive pathway for PEDV internalization.

### Productive endocytosis of PEDV cooperatively mediated by clathrin.

**FIG 6:**
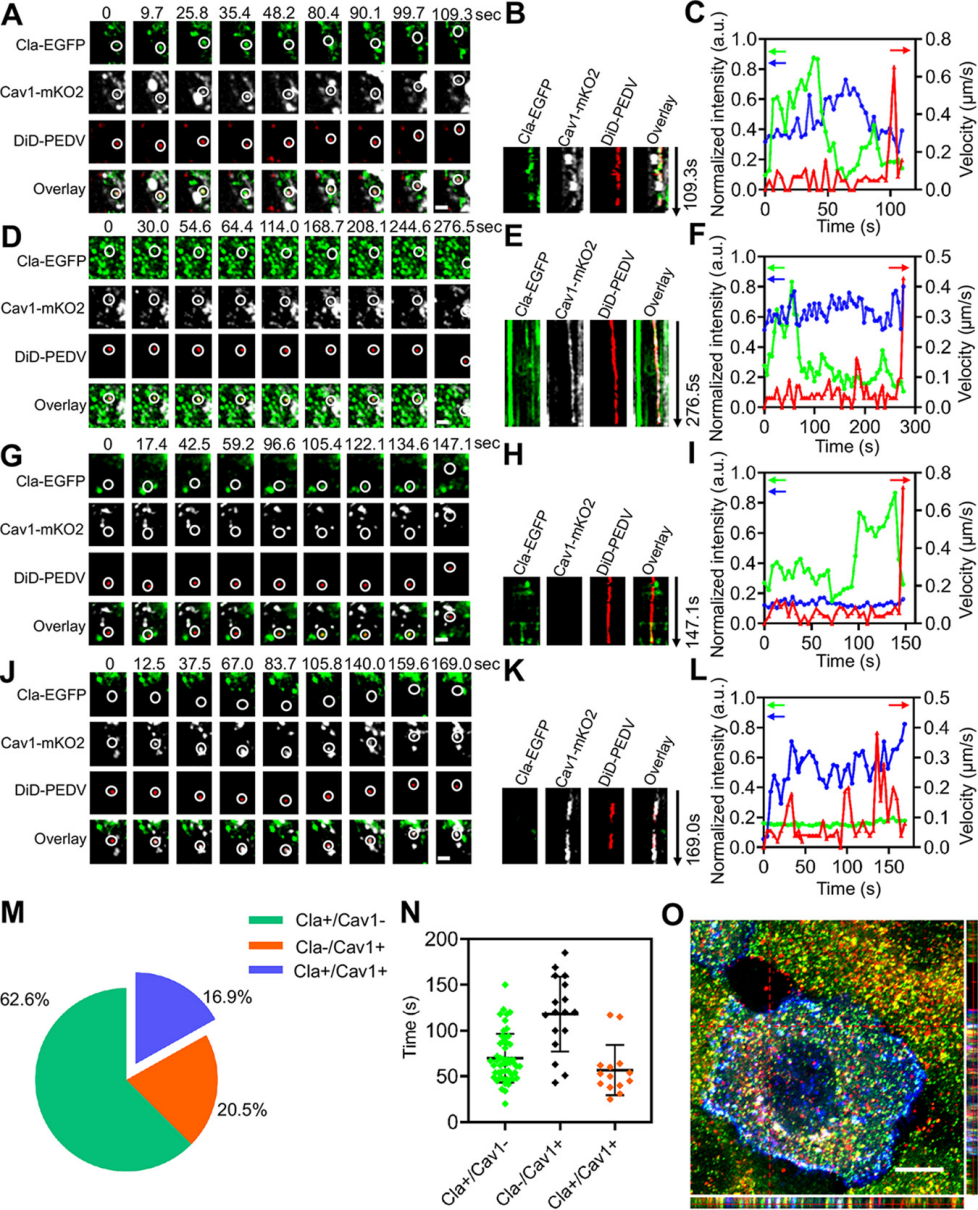
Productive C^3^ME of PEDV. (A to C) PEDV infection via C^3^ME captured by triple-color confocal microscope. (A) Time-lapse images of PEDV (red) entering cells coexpressing Cla-EGFP (green) and Cav1-mKO2 (white). Scale bar, 2 μm. (B) Kymographs at viral binding site. (C) Time-lapse clathrin and caveolin-1 fluorescence intensities at the viral binding site and PEDV velocity. (D to F) PEDV infection via C^3^ME captured by triple-color TIRF microscope. (D) Time-lapse images of PEDV (red) entering cells coexpressing Cla-EGFP (green) and Cav1-mKO2 (white). Scale bar, 2 μm. (E) Kymographs at the viral binding site. (F) Time-lapse clathrin and caveolin-1 fluorescence intensities at the viral binding site and PEDV velocity. (G to I) PEDV infection via CME captured by triple-color confocal microscope. (G) Time-lapse images of PEDV (red) entering cells coexpressing Cla-EGFP (green) and Cav1-mKO2 (white). Scale bar, 2 μm. (H) Kymographs at viral binding site. (I) Time-lapse clathrin and caveolin-1 fluorescence intensities at the viral binding site and PEDV velocity. (J to L) PEDV infection via CavME captured by triple-color confocal microscope. (J) Time-lapse images of PEDV (red) entering cells coexpressing Cla-EGFP (green) and Cav1-mKO2 (white). Scale bar, 2 μm. (K) Kymographs at the viral binding site. (L) Time-lapse clathrin and caveolin-1 fluorescence intensities at the viral binding site and PEDV velocity. (M) Proportion of PEDVs entering cells via CME (*n* = 52), CavME (*n* = 17), and C^3^ME (*n* = 14). (N) Duration in different pathways; error bars represent SD (*n* = 83). (O) 3D fluorescence colocalization imaging on CCP, caveolae, and PEDV using confocal microscope. Scale bar, 10 μm.

We found PEDVs entered cells not only via CME or CavME alone but also when clathrin and caveolae cooperatively mediated endocytosis (C^3^ME), in which viruses can simultaneously utilize both CCPs and caveolae to enter cells. Using single-virus tracking relying on a triple-color confocal microscope, the dynamics of PEDV entering into cells coexpressing Cla-EGFP and Cav1-mKO2 were observed (Fig. 6A; see Movie S3a in the supplemental material). Colocalization among PEDV, CCP, and caveolae, as shown in kymographs of time-lapse images at the viral binding site (Fig. 6B), proves that PEDV entered the cell via C^3^ME. Moreover, PEDV velocity, Cla-EGFP and Cav1-mKO2 fluorescence intensity, and trajectory (see Fig. S2A in the supplemental material) reveal that there were still two stages in PEDV internalization via C^3^ME. First, in the viral binding stage, PEDV in caveolae recruited clathrin proteins, a process revealed by increased Cla-EGFP fluorescence intensity and relatively stable Cav1-mKO2 fluorescence intensity with low speed and restricted movement shown in (Fig. 6C and Fig. S2B). Second, in the viral moving stage, the PEDV entered the cell with accelerated speed and directed movement (Fig. 6C; Fig. S2C) accompanied by decreased Cla-EGFP fluorescence intensity and stationary Cav1-mKO2 fluorescence intensity.

10.1128/mBio.00256-21.2 FIG S2 Kinetic analysis on single PEDV particles entering cells coexpressing Cla-EGFP and Cav1-mKO2. (A to C, D to F, G to I, J to L) Trajectories of PEDV infection during viral binding (green) and moving (red) stages, and MSD plots of the PEDV movements during viral binding and moving stages, respectively. Download FIG S2, TIF file, 0.3 MB. Copyright © 2021 Li et al. 2021 Li et al. https://creativecommons.org/licenses/by/4.0/ This content is distributed under the terms of the Creative Commons Attribution 4.0 International license.

10.1128/mBio.00256-21.5 MOVIE S3 (a) Productive endocytosis of PEDV (red) via C^3^ME captured by triple-color confocal microscope in Vero CCL81 cells coexpressing Cla-EGFP (green) and Cav1-mKO2 (white), corresponding to the time-lapse images in Fig. 6A. (b) Productive endocytosis of PEDV (red) via C^3^ME captured by triple-color TIRF microscope in Vero CCL81 cells coexpressing Cla-EGFP (green) and Cav1-mKO2 (white), corresponding to the time-lapse images in Fig. 6D. (c) Productive endocytosis of PEDV (red) via CME captured by triple-color TIRF microscope in Vero CCL81 cells coexpressing Cla-EGFP (green) and Cav1-mKO2 (white), corresponding to the time-lapse images in Fig. 6G. (d) Productive endocytosis of PEDV (red) via CavME captured by triple-color TIRF microscope in Vero CCL81 cells coexpressing Cla-EGFP (green) and Cav1-mKO2 (white), corresponding to the time-lapse images in Fig. 6J. Download Movie S3, MOV file, 0.9 MB. Copyright © 2021 Li et al. 2021 Li et al. https://creativecommons.org/licenses/by/4.0/ This content is distributed under the terms of the Creative Commons Attribution 4.0 International license.

Moreover, in order to determine where C^3^ME of PEDV occurred, single-virus tracking using a triple-color total internal reflected fluorescence (TIRF) microscope was adopted to record time-lapse images of PEDV entering cells via C^3^ME (Fig. 6D; Movie S3b). Based on the colocalization among PEDV, CCP, and caveolae in kymographs of time-lapse images at the viral binding site (Fig. 6E), as well as the similar PEDV velocity (Fig. 6F), trajectory (Fig. S2D), movements (see Fig. S2E and F in the supplemental material), and Cla-EGFP and Cav1-mKO2 fluorescence signal variations (Fig. 6F) compared with previous results (Fig. 2 and 3), it is clear that PEDV entered the cell via C^3^ME. TIRF microscopes can collect only fluorescence signals near the cell membrane (22); therefore, we believe that C^3^ME of PEDV occurred near the cell membrane.

Besides C^3^ME of PEDV, CME and CavME of PEDV can also be observed using single-virus tracking relying on triple-color TIRF (Fig. 6G to L; Fig. S2G to L; Movie S3c and d). It is worth noting that PEDV movements, as well as Cla-EGFP and Cav1-mKO2 fluorescence signal variations, are similar to previous results (Fig. 2 and 3). Moreover, a total of 83 viral productive endocytosis events were observed; among them, 62.6% of PEDVs entered cells via CME, 20.5% via CavME, and 16.9% via C^3^ME (Fig. 6M), indicating that PEDV prefers recruiting clathrin proteins for cell entry. The duration of CCPs, caveolae, and clathrin/caveolin-1 double-positive vesicles was also analyzed (Fig. 6N), and the results are as follows: the duration of CCPs in cases of productive CME is 56.9 ± 27.5 s, that of caveolae in cases of productive CavME is 118.1 ± 41.0 s, and that of CCPs and caveolae in cases of productive C^3^ME is 69.9 ± 26.5 s. The duration of CCPs and caveolae in different pathways indicates that the recruitment of clathrin proteins may promote the entry of PEDVs into cells. Additionally, three-dimensional (3D) fluorescence imaging was also used to validate the colocalization among PEDV, CCP, and caveolae (Fig. 6O), providing further evidence for C^3^ME of PEDV.

**FIG 7:**
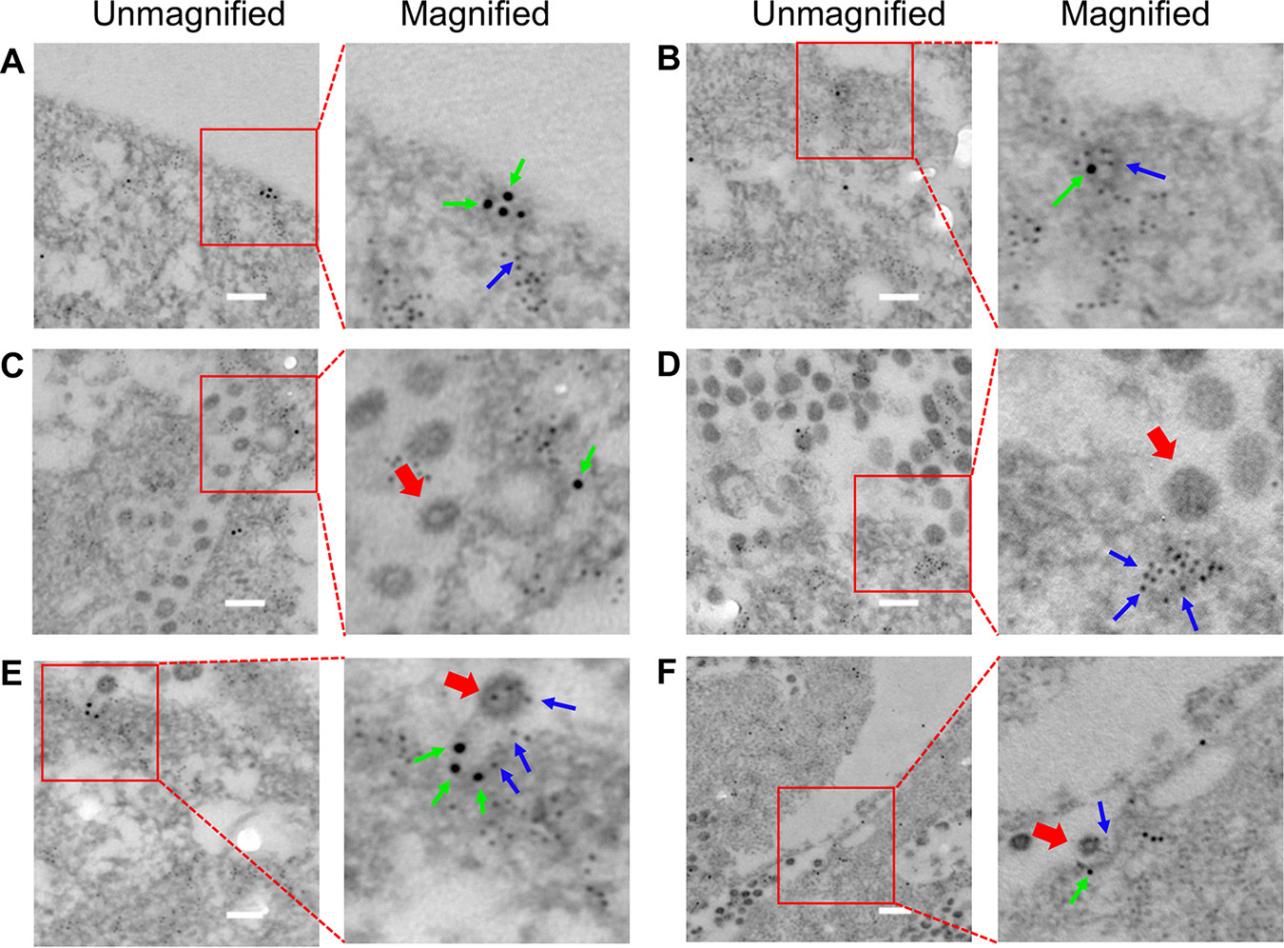
Clathrin and caveolin-1 double immunogold electron microscopy of PEDV entry pathway. (A) Distinct regions of CCPs and caveolae on cell membrane. (B) Clathrin and caveolin-1 double-positive invagination on cell membrane. (C) CME of PEDV. (D) CavME of PEDV. (E, F) C^3^ME of PEDV. Clathrin was labeled with 10-nm immunogold, as indicated by the green arrow; caveolin-1 was labeled with 4-nm immunogold, as indicated by the blue arrow; and PEDV was indicated by the red arrow. Scale bar, 200 nm.

Moreover, clathrin and caveolin-1 double immunogold electron microscopy (EM) was used to reveal the entry pathway of PEDV. The results demonstrated the existence of not only distinct CCP and caveolae regions on the cell membrane (Fig. 7A) but also clathrin and caveolin-1 double-positive invagination on the cell membrane in PEDV-infected cells (Fig. 7B). The dual immunogold EM results also clearly exhibited that PEDV could induce the formation of CCPs (Fig. 7C), caveolae (Fig. 7D), and clathrin and caveolin-1 double-positive invaginations (Fig. 7E and F) on the cell membrane, indicating CME, CavME, and C^3^ME of PEDV, respectively. The results obtained from single-virus tracking, 3D fluorescence imaging, and dual immunogold electron microscopy all proved that CME, CavME, and C^3^ME existed simultaneously in productive PEDV internalization. To our best knowledge, this is the first work reporting that viruses can exploit C^3^ME to enter cells, and this viral entry manner is completely different from the reported CME and CavME.

### Abortive internalization of PEDV.

**FIG 8:**
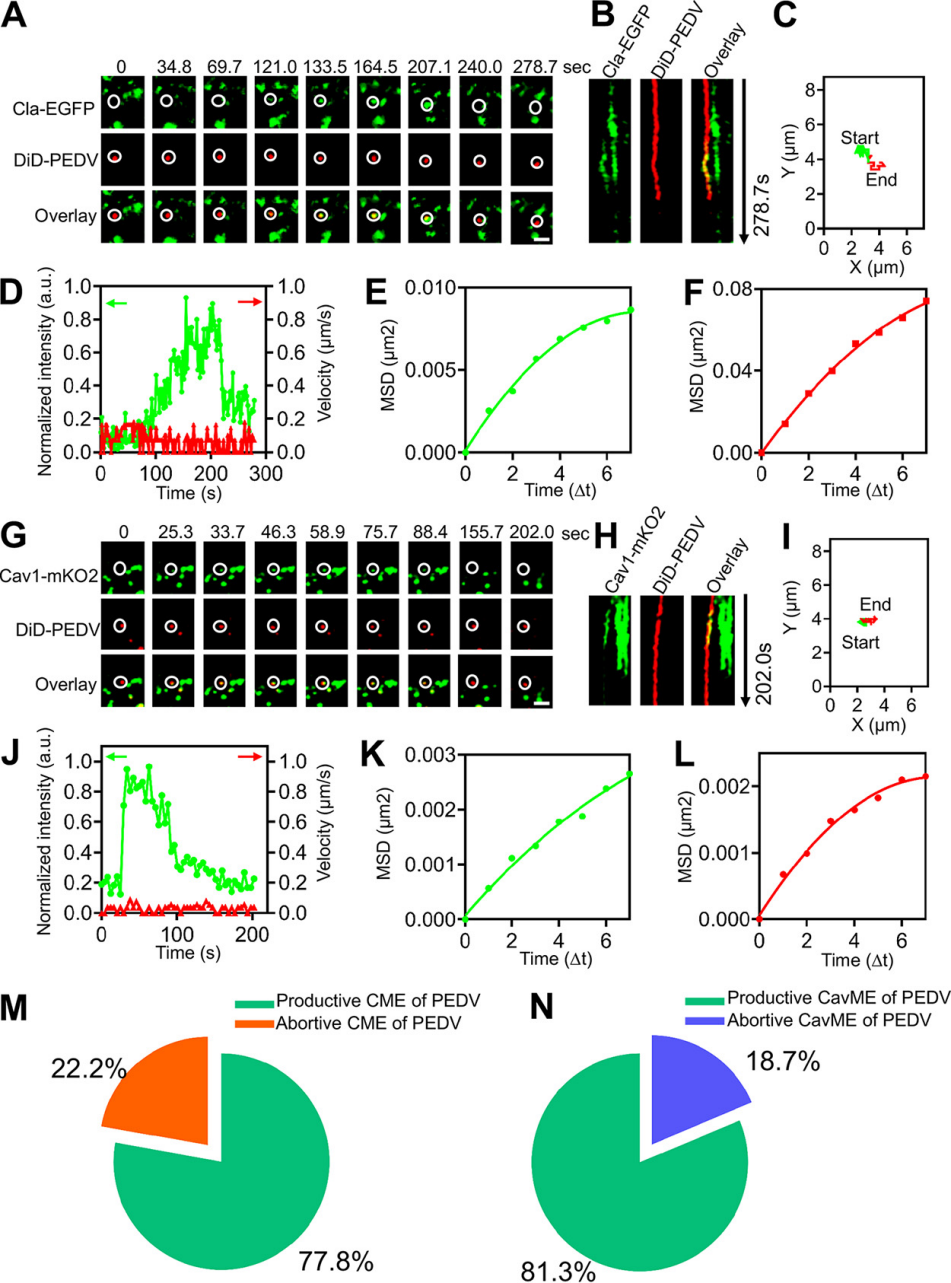
Abortive internalization of PEDV. (A to F) Abortive internalization of PEDV via CME. (A) Time-lapse images of PEDV (red)-infected cells expressing Cla-EGFP (green). Scale bar, 2 μm. (B) Kymographs at the viral binding site. (C) Trajectory of PEDV infection. (D) Time-lapse clathrin fluorescence intensity at the viral binding site and velocity of PEDV. (E, F) MSD plots of the PEDV movements during viral diffusion. (G to L) Abortive internalization of PEDV via CavME. (G) Time-lapse images of PEDV (red)-infected cells expressing Cav1-mKO2 (green). Scale bar, 2 μm. (H) Kymographs at the viral binding site. (I) Trajectory of PEDV infection. (J) Time-lapse clathrin fluorescence intensity at the viral binding site and velocity of PEDV. (K, L) MSD plots of the PEDV movements during viral diffusion. (M) Proportion of the productive (*n* = 35) and abortive (*n* = 10) internalization of PEDVs recruiting clathrin. (N) Proportion of the productive (*n* = 26) and abortive (*n* = 6) internalization of PEDVs recruiting caveolin-1.

Besides productive internalization of PEDV via CME, CavME, and C^3^ME, we also found that some PEDVs could successfully induce CCPs or caveolae, but they did not enter cells and instead remained at the cell surface after the disappearance of the CCPs or caveolae. This scenario was probed by single-virus tracking (Fig. 8). Based on the dynamic recruitment of clathrin and caveolae structures and viral motility, these events are defined as abortive internalization of PEDV. Abortive CME and CavME of PEDVs were both observed (Fig. 8A and G; see Movie S4a and b in the supplemental material). Kymographs of the time-lapse images (Fig. 8B and H) indicate colocalization between PEDV and CCP, as well as between PEDV and caveolae, proving the PEDVs recruited CCPs or caveolae. But, even after Cla-EGFP or Cav1-mKO2 fluorescence signals disappeared (Fig. 8D and J), the PEDVs remained at the cell surface, as revealed by the trajectories (Fig. 8C and I), and exhibited extremely low speed (Fig. 8D and J) and restricted movement (Fig. 8F and L). However, we did not observe abortive internalization when PEDVs recruited both CCPs and caveolae. Moreover, our statistical analysis of the abortive internalization of PEDV showed that 22.2% of PEDVs (10/45 in 25 cells) underwent abortive CME and 18.7% (6/32 in 25 cells) underwent abortive CavME (Fig. 8M and N). These results suggest that there is still abortive internalization of PEDV via CME and CavME in addition to productive internalization.

10.1128/mBio.00256-21.6 MOVIE S4 (a) Abortive endocytosis of PEDV (red) via CME in Vero CCL81 cells expressing Cla-EGFP (green), corresponding to the time-lapse images in Fig. 7A. (b) Abortive endocytosis of PEDV (red) via CavME in Vero CCL81 cells expressing Cav1-mKO2 (green), corresponding to the time-lapse images in Fig. 7G. Download Movie S4, MOV file, 0.7 MB. Copyright © 2021 Li et al. 2021 Li et al. https://creativecommons.org/licenses/by/4.0/ This content is distributed under the terms of the Creative Commons Attribution 4.0 International license.

### PEDV trafficking from early to late endosomes.

**FIG 9:**
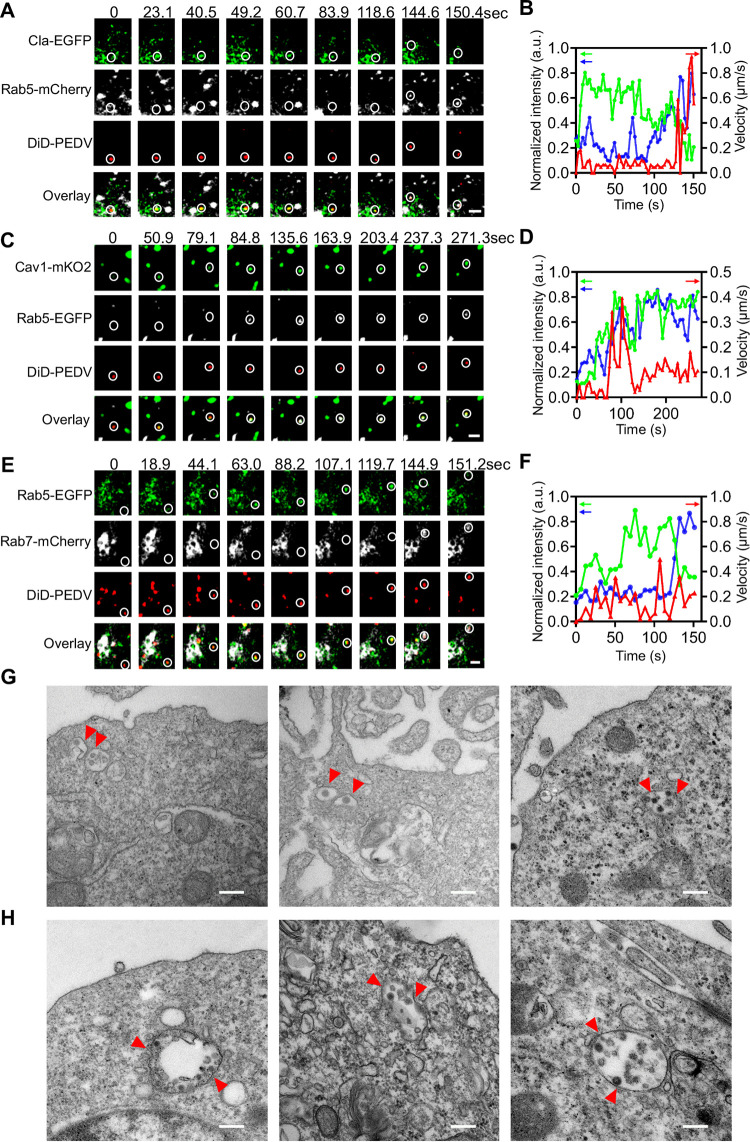
PEDV trafficking from early to late endosomes. (A) Time-lapse images of PEDV (red) via CME from CCP (green) to Rab5 positive early endosome (white). Scale bar, 2 μm. (B) Time-lapse Cla-EGFP (green) and Rab5-mCherry (blue) fluorescence intensities at viral binding site and PEDV velocity (red) corresponding to (A). (C) Time-lapse images of PEDV (red) via CavME from caveolae (green) to Rab5 positive early endosome (white). Scale bar, 2 μm. (D) Time-lapse Cav1-mKO2 (green) and Rab5-EGFP (blue) fluorescence intensities at viral binding site and PEDV velocity (red) corresponding to (C). (E) Time-lapse images of PEDV (red) transporting from early endosome (green) to late endosome (white). Scale bar, 2 μm. (F) Time-lapse Rab5-EGFP (green) and Rab7-mCherry (blue) fluorescence intensities at viral binding site and PEDV velocity (red) corresponding to (E). (G, H) Ultrastructural analysis on the internalization of PEDV particles in endosomes. Scale bar, 200 nm.

We found that PEDV was directly delivered to endosomes after CME or CavME, which was also revealed using single-virus tracking. According to the time-lapse images of PEDV trafficking after CME of PEDV (Fig. 9A; see Movie S5a in the supplemental material), as Cla-EGFP fluorescence intensity decreased, PEDV accelerated to reach the early endosome with increased Rab5-mCherry fluorescence intensity (Fig. 9B). Moreover, based on the time-lapse images of PEDV trafficking after CavME of PEDV (Fig. 9C; Movie S5b), PEDV accelerated to reach the early endosome with increased Rab5-mCherry fluorescence intensity but with nearly stable Cav1-mKO2 fluorescence intensity (Fig. 9D). These results prove that PEDVs entered into early endosomes after internalization.

10.1128/mBio.00256-21.7 MOVIE S5 (a) PEDV (red) trafficking from CCPs to early endosomes in Vero CCL81 cells coexpressing Cla-EGFP (green) and Rab5-mCherry (white), corresponding to the time-lapse images in Fig. 8A. (b) PEDV (red) trafficking from caveolae to early endosomes in Vero CCL81 cells coexpressing Cav1-mKO2 (green) and Rab5-mCherry (white), corresponding to the time-lapse images in Fig. 8C. (c) PEDV (red) trafficking from early endosomes to late endosomes in Vero CCL81 cells coexpressing Rab5-EGFP (green) and Rab7-mCherry (white), corresponding to the time-lapse images in Fig. 8E. Download Movie S5, MOV file, 0.6 MB. Copyright © 2021 Li et al. 2021 Li et al. https://creativecommons.org/licenses/by/4.0/ This content is distributed under the terms of the Creative Commons Attribution 4.0 International license.

We also observed using single-virus tracking that PEDVs were transported from early endosomes to late endosomes (Fig. 9E; Movie S5c). Time-lapse images show that Rab5-EGFP fluorescence intensity decreased while Rab7-mCherry fluorescence intensity increased (Fig. 9F). These results verify PEDV trafficking from Rab5-positive early endosomes to Rab7-positive late endosomes.

**FIG 10:**
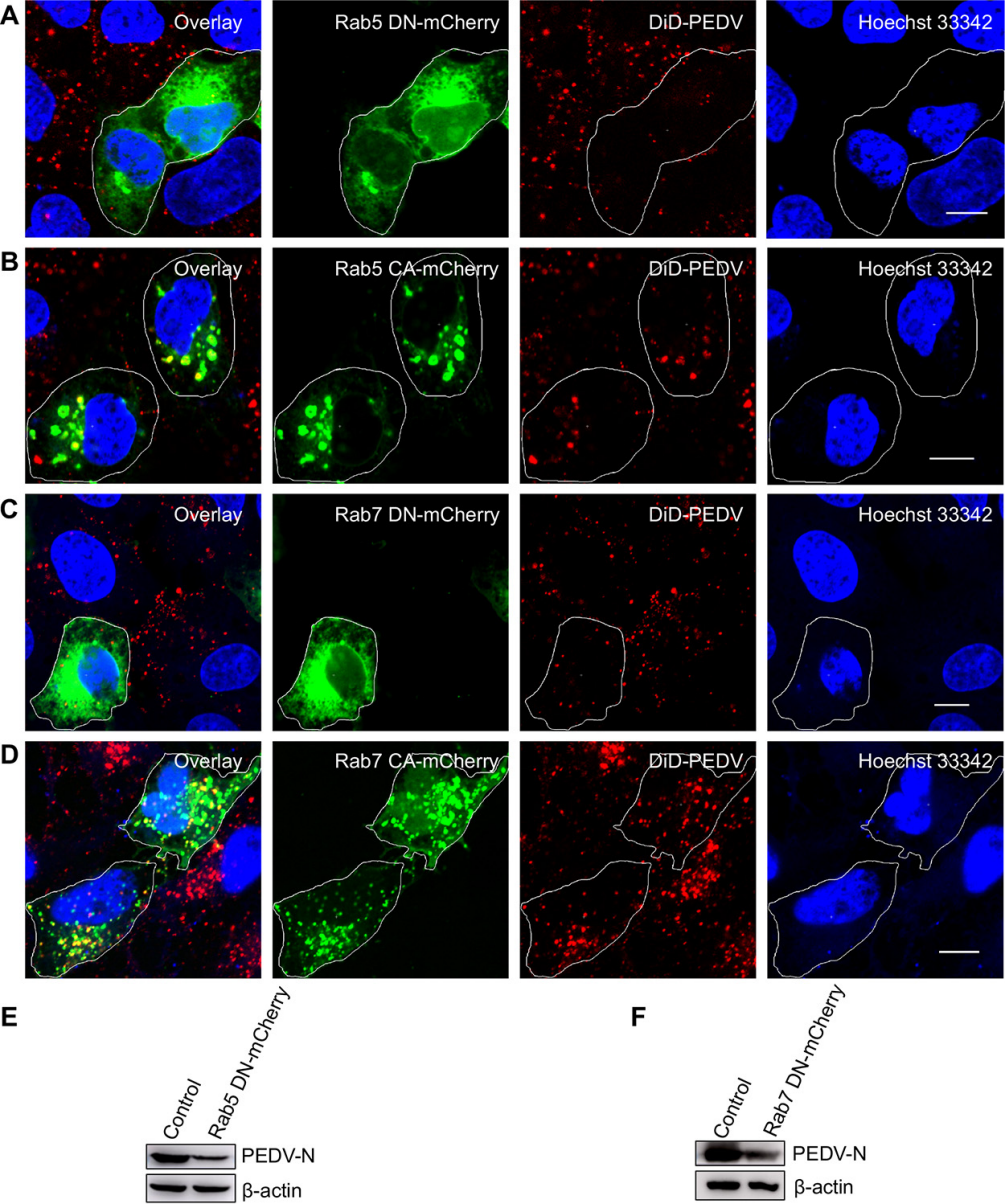
The infection of PEDV is dependent on early and late endosomes. (A, B) Fluorescence images of the infection of DiD-labeled PEDVs in cells expressing Rab5 CA-mCherry or Rab5 DN-mCherry. After 24 h of transfection, cells were infected with DiD-labeled PEDVs (MOI, 10) for 30 min, and then samples were fixed and imaged. (C, D) Fluorescence images of the infection of DiD-labeled PEDVs in cells expressing Rab7 CA-mCherry or Rab7 DN-mCherry. After 24 h of transfection, cells were infected with DiD-labeled PEDVs (MOI, 10) for 30 min, and then samples were fixed and imaged. (E, F) Cells were transfected with plasmid Rab5 DN-mCherry or Rab7 DN-mCherry. After 24 h of transfection, cells were infected with PEDVs (MOI, 1) and cultured for 6 h. Then, samples were collected and analyzed by Western blotting with corresponding antibodies. PEDV-infected untransfected cells served as controls. Scale bar, 10 μm.

In addition, we used TEM to investigate PEDV endosome trafficking at an ultrastructural level (Fig. 9G and H). PEDV particles were compassed in the endosomes. To further confirm that the PEDV endosome trafficking is Rab5 and Rab7 dependent, we used Rab5CA and Rab5DN to disturb early endosomes and Rab7CA and Rab7DN to disturb late endosomes. In cells expressing Rab5DN and Rab7DN, PEDV entry was remarkably reduced (Fig. 10A and C). In contrast, in cells expressing Rab5CA and Rab7CA, PEDVs could be enriched only in early endosomes (Fig. 10B and D). The results of Western blotting revealed that Rab5DN and Rab7DN can inhibit PEDV N protein expression in PEDV infection (Fig. 10E and F).

The PEDV endosome trafficking observed by single-virus tracking and results obtained from TEM and inhibition experiments prove that PEDVs entered early endosomes after CME and CavME and were then transported from early endosomes to late endosomes.

### PEDV fusion.

**FIG 11:**
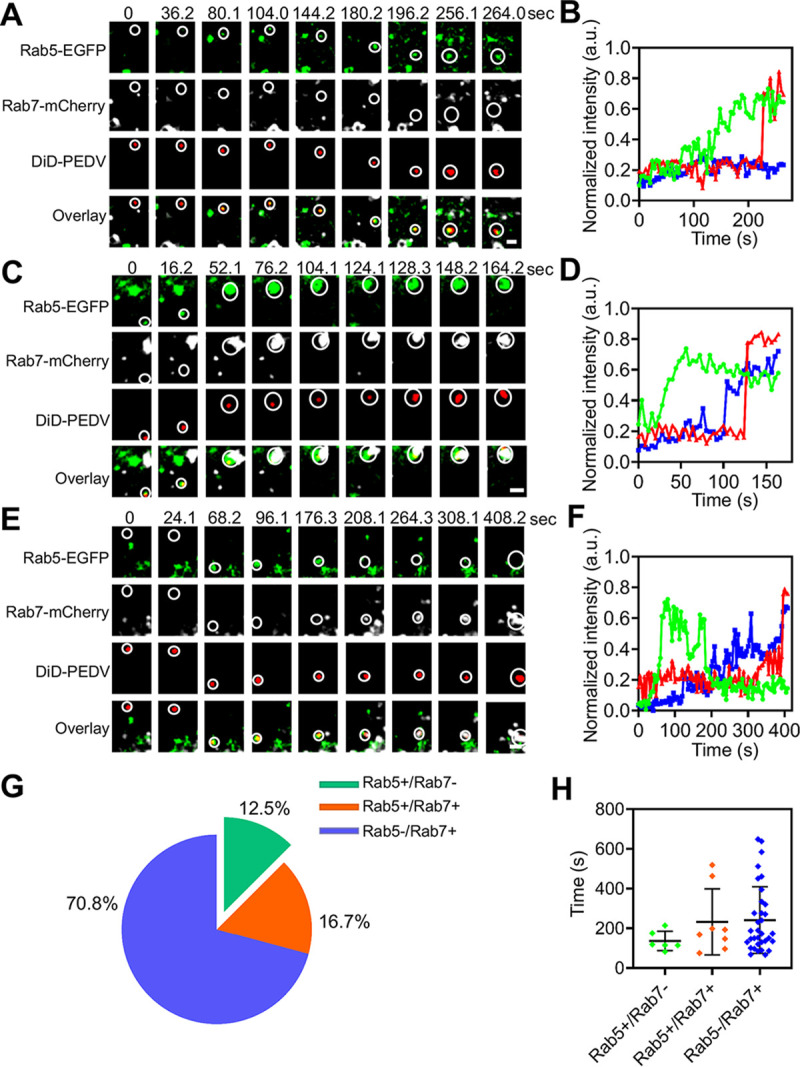
PEDV fusion. (A) Time-lapse images of PEDV (red) fused in Rab5-positive early endosome (green). Scale bar, 2 μm. (B) Time-lapse Rab5-EGFP (green), Rab7 (white), and DiD-labeled PEDV (red) fluorescence intensities corresponding to A. (C) Time-lapse images of PEDV (red) fused in Rab5 (green) and Rab7 (white) double-positive endosome. Scale bar, 2 μm. (D) Time-lapse Rab5 (green), Rab7 (blue,) and DiD-labeled PEDV (red) fluorescence intensities corresponding to C. (E) Time-lapse images of PEDV (red) fused in Rab7-positive late endosome (white). Scale bar, 2 μm. (F) Time-lapse Rab5 (green), Rab7 (blue), and DiD-labeled PEDV (red) fluorescence intensities corresponding to E. (G) Proportion of PEDV fusion with Rab5-positive (*n* = 6), Rab5 and Rab7 double positive (*n* = 8), and Rab7-positive (*n* = 34) endosomes. (H) Colocalization time of PEDV with Rab5-positive, Rab5 and Rab7 double positive, and Rab7-positive endosomes before viral fusion. Error bars represent SD.

Finally, PEDV fusion was investigated using single-virus tracking. When adequate DiD dyes incorporate into the envelope of virions, the DiD fluorescence is self-quenched but still allows detection of a single DiD-labeled virion (23, 24). Therefore, when the envelope of a DiD-PEDV particle fuses with the endosomal membrane, DiD dyes are fluorescently dequenched, leading to a significant increase of fluorescence intensity. PEDV fusion occurred in the early endosome since DiD fluorescence intensity dramatically increased with high Rab5-EGFP fluorescence intensity but low Rab7-mCherry fluorescence intensity (Fig. 11A and B; see Movie S6a in the supplemental material). Additionally, PEDV fusion occurred in the intermediate endosome because DiD fluorescence intensity dramatically increased with both high Rab5-EGFP and Rab7-mCherry fluorescence intensities (Fig. 11C and D; Movie S6b). PEDV fusion also occurred in the late endosome, as DiD fluorescence intensity dramatically increased with low Rab5-EGFP fluorescence intensity but high Rab7-mCherry fluorescence intensity (Fig. 11E and F; Movie S6c). Therefore, PEDV fusion can occur in the early, intermediate, and late endosomes. A total of 48 viral fusion events were observed via single-virus tracking; among them, 12.5% of PEDVs fused in early endosomes, 16.7% in intermediate endosomes, and 70.8% in late endosomes (Fig. 11G), illustrating that most PEDV fusion occurred in late endosomes.

10.1128/mBio.00256-21.8 MOVIE S6 (a) PEDV (red) fusion in early endosomes in Vero CCL81 cells coexpressing Rab5-EGFP (green) and Rab7-mCherry (white), corresponding to the time-lapse images in Fig. 9A. (b) PEDV (red) fusion in intermediate endosomes in Vero CCL81 cells coexpressing Rab5-EGFP (green) and Rab7-mCherry (white), corresponding to the time-lapse images in Fig. 9C. (c) PEDV (red) fusion in late endosomes in Vero CCL81 cells coexpressing Rab5-EGFP (green) and Rab7-mCherry (white), corresponding to the time-lapse images in Fig. 9E. Download Movie S6, MOV file, 0.8 MB. Copyright © 2021 Li et al. 2021 Li et al. https://creativecommons.org/licenses/by/4.0/ This content is distributed under the terms of the Creative Commons Attribution 4.0 International license.

We also analyzed viral fusion kinetics by measuring the colocalization time between PEDVs and endosomes (Fig. 11H). In Rab5-positive endosomes (early endosomes), PEDV exhibited 136.4- ± 48.5-s virus-endosome colocalization time before viral fusion occurred. In Rab5 and Rab7 double-positive endosomes (intermediate endosomes), PEDV exhibited 232.4- ± 165.9-s virus-endosome colocalization time before viral fusion occurred. In Rab7-positive endosomes (late endosomes), PEDV exhibited 241.2- ± 167.9-s virus-endosome colocalization time before viral fusion occurred. These data indicate that PEDV exhibited shorter virus-endosome colocalization time in the early endosomes than in the intermediate and late endosomes before PEDV fusion occurred in the different stages of endosomes.

Moreover, our results also showed that 87.5% of PEDV particles dequenched in Rab7-positive endosomes (intermediate and late endosomes), indicating that the majority of PEDV particles were transported to the Rab7-positive compartments for viral fusion. These data may also imply that a longer duration of acid accumulation of the Rab7-positive compartments is one of the main triggers for PEDV membrane fusion since the pH decreases progressively during endosomal maturation (25, 26).

## DISCUSSION

When confronting host cells, enveloped viruses have evolved a variety of pathways to deliver viral genomes into the cytoplasm to initiate their life cycle (26–28). Unraveling how viruses penetrate cell membrane barriers is not only important for deepening our understanding of viral infection but also helpful for designing antiviral drugs. In this study, we mainly used single-virus tracking in living cells to dynamically reveal the early stages of PEDV infection, including the endocytic pathway, endosome trafficking, and viral fusion, which were previously not fully understood.

Hijacking the endocytic pathway is critical for viruses to penetrate the cell membrane barrier. CoVs, like other viruses, are obligate parasites which depend on host cell factors for propagation. It is an emerging theme that the same virus might infect cells with a lack of strict dependence of one pathway to make a productive infection. Several CoVs, such as severe acute respiratory syndrome coronavirus (SARS-CoV), mouse hepatitis coronavirus (MHV), human coronavirus OC43 (HCoV-OC43) and infectious bronchitis coronavirus (IBV), have been demonstrated to use multiple pathways to enter cells (29–32). Previous studies showed that PEDV enters cells via CME, whereas the CavME inhibitor methyl-β-cyclodextrin could also significantly inhibit PEDV infection in the pre-entry period, indicating that CavME may also be a pathway for PEDV entry (14). We directly observed the *de novo* formation of CCPs and caveolae at the viral binding sites in CME and CavME of PEDV, respectively. This finding is consistent with a very recent study showing that PEDV can enter cells via CME and CavME (14). Moreover, an analysis of the viral velocities and mean square displacement (MSD) revealed that PEDVs first moved slowly at plasma membranes with restricted diffusion and then moved rapidly into the cytoplasm with directed diffusion, indicating that PEDV enters into cells with different diffusion patterns. In addition, we also directly visualized CME and CavME at an ultrastructural level using TEM. To further confirm the roles of CME and CavME, inhibition experiments proved that cells transfected with Eps15-DN, Cav1-DN, or Cav3-DN significantly suppressed PEDV internalization. Single-virus tracking, TEM, and inhibition experiments illustrated that CME and CavME are two productive pathways for PEDV entry.

Although CME and CavME are distinct endocytic pathways, these two pathways are not absolutely irrelevant. For example, bovine papillomavirus type 1 (BPV1) can enter cells via a clathrin-dependent pathway and then utilize a caveolae-dependent pathway for infection, revealing a pattern that viruses are from CCPs to caveolae (33). Besides, the direct fusion of CCPs and caveolae is also found during the internalization of TGF-β receptors; and the clathrin and caveolae double-positive vesicles appear half smooth and half fuzzy when visualized by TEM (34). In this work, we used triple-color confocal and TIRF microscopes to not only confirm independent CME and CavME of PEDV but also reveal C^3^ME of PEDV. Similar to CME and CavME, in C^3^ME, PEDVs first moved slowly at the plasma membrane with restricted diffusion and then moved rapidly into the cytoplasm with directed diffusion. Among the observed productively internalized PEDVs, 62.6% entered cells via CME, 20.5% via CavME, and 16.9% via C^3^ME. It can also be inferred by the duration of CCPs and caveolae that the duration of CCPs in CME and that of CCPs and caveolae in C^3^ME are obviously shorter than that of caveolae in CavME. An analysis of the proportion of PEDV entry route and duration indicates that, compared to caveolae recruitment, CCP recruitment is more beneficial for PEDV internalization. Moreover, the average time of PEDV penetrating the cell membrane was almost within ∼3 min, suggesting that viral particles may require a short time to recruit receptors or cofactors, once the formation of CCPs and caveolae at viral binding sites, the entry process of PEDV is extremely fast. Also, the dual immunogold EM results clearly exhibited that PEDV could induce the formation of CCPs, caveolae, and clathrin and caveolin-1 double-positive invaginations on the cell membrane, indicating CME, CavME, and C^3^ME of PEDV, respectively. C^3^ME of PEDV was also validated by colocalization among PEDVs, CCPs, and caveolae using 3D imaging. Together, our results revealed a novel manner of CoV entry that entails the cooperation of multiple endocytic pathways, such as C^3^ME, which may also exist in other CoVs.

In addition to productive PEDV internalization via CME, CavME, and C^3^ME, abortive PEDV internalization via CME and CavME was also observed using single-virus tracking. These PEDVs had consistently slow movement at the plasma membrane, with restricted diffusion even after CCPs or caveolae disappeared. These results provided direct evidence that not all hijacking of the host cell endocytic pathways by viruses can make a productive infection. Similar abortive endocytosis events were also reported for which influenza virus induced the abortive recruitments of clathrin and dynamin (35). Note that we observed abortive PEDV internalization via CME or CavME but not via C^3^ME, implying that the C^3^ME manner may be a more efficient pathway for PEDV infection. Moreover, our statistical analysis of the abortive internalization of PEDV shows that about 20% of PEDVs experienced abortive endocytosis, suggesting that the majority of PEDVs can hijack the endocytic pathways to make a productive infection.

It is known that the early endosome is the initial station for cellular cargo sorting (36). Most viruses, such as the Semliki Forest virus (SFV), chikungunya virus, and severe fever with thrombocytopenia syndrome virus (SFTSV) are first delivered to early endosomes and then transported to late endosomes (37–39). We directly observed that PEDV moved from clathrin structures or caveolae structures to early endosomes. After leaving clathrin structures or caveolae structures, PEDVs entered early endosomes with accelerated speed, exhibiting the colocalization between DiD-labeled PEDVs and Rab5 early endosomes, and then were transported to late endosomes, shown by colocalization between DiD-labeled PEDVs and Rab7 late endosomes. Moreover, the transfection of constitutively active mutants Rab5a Q79L and Rab7a Q67L resulted in colocalization between DiD-labeled PEDVs and Rab5 early endosome/Rab7 late endosome, respectively; and transfection of the dominant-negative mutants Rab5a S34N and Rab7a T22N significantly reduced PEDV entry. Both of these results prove that early and late endosomes are involved in PEDV entry.

Additionally, viral fusion is a crucial step in PEDV entry. We directly observed PEDV fusion in endosomes via single-virus tracking. Although, PEDV fusion occurred in early, intermediate, and late endosomes, statistical analysis shows that PEDV predominantly (70.8%) fused in late endosomes within ∼6.8 min after the transport of PEDV to late endosomes. Our results clearly show that PEDV was colocalized with late endosomes several minutes before the onset of membrane fusion, implying that membrane fusion of PEDV might require other host cell factors, such as lysosome, and the detailed membrane fusion mechanism of PEDV still needs to be further investigated.

**FIG 12:**
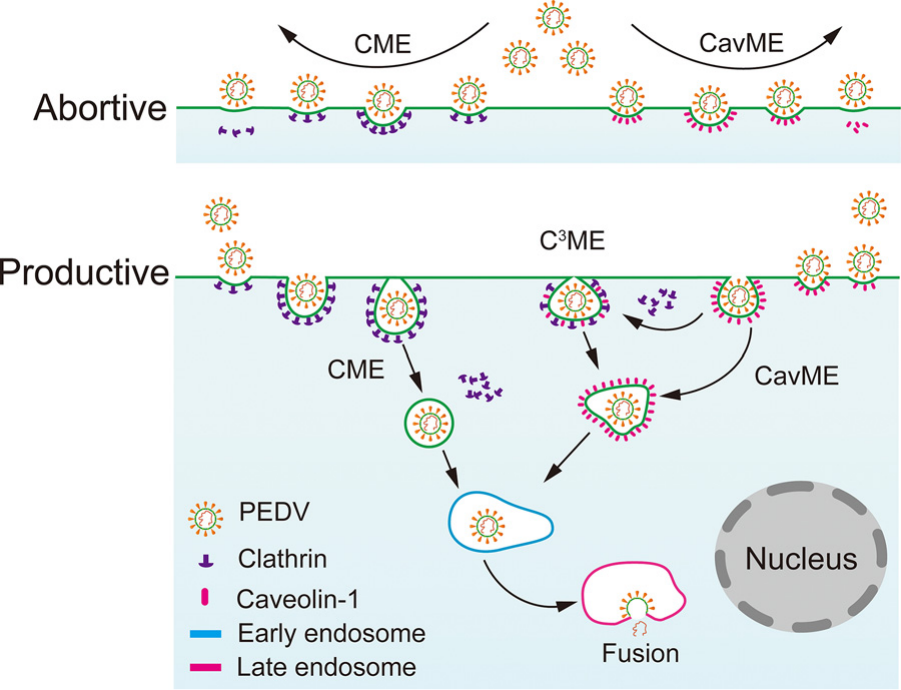
Entry model of PEDV into host cells. PEDV infection started with the recruitment of clathrin and caveolin-1. About 20% PEDVs experienced abortive internalization. Productive internalized PEDVs hijacked CME, CavME, and C^3^ME to penetrate cell membrane and then were delivered to early and late endosomes. Finally, PEDV fusion mainly occurred in the late endosomes.

In this work, using single-virus tracking, we systematically unraveled the dynamic interactions of individual PEDVs, clathrin structures, caveolae, and early and late endosomes. Hence, we revealed the C^3^ME mechanism of PEDV that during CavME of PEDV, clathrin structures can fuse with caveolae near the cell plasma membrane to make a productive infection, and with about a proportion of 20%, PEDV underwent an abortive endocytosis via CME or CavME. Moreover, trafficking of PEDV from clathrin and caveolae structures to early endosomes and from early endosomes to late endosomes was revealed. Also, PEDV fusion mainly occurred in late endosomes within ∼6.8 min after the transport of PEDV to late endosomes (Fig. 12). In conclusion, we dissected the dynamic PEDV entry in living cells at the single-virus level. The cooperation of multiple endocytic pathways, such as C^3^ME, may also exist in other coronaviruses, which not only expands our understanding of the mechanism of CoV infection, but also may benefit the development of potential anticoronavirus therapeutic strategies.

## MATERIALS AND METHODS

### Cell culture, virus propagation, and purification.

First, Vero cells (ATCC CCL-81) were cultured in Dulbecco’s modified Eagle medium (DMEM; Gibco, USA), supplemented with 10% fetal bovine serum (FBS; Biological Industry, Israel) and antibiotics (100 U/ml penicillin and 100 μg/ml streptomycin), and maintained in a humidified atmosphere containing 5% CO_2_ at 37°C. Then, PEDV strain CV777 (provided by Gang Xing, Zhejiang University, China) was propagated in Vero cells in FBS-free DMEM containing 0.3% tryptose phosphate broth (TPB; Sigma, USA) and 3 μg/ml trypsin (Sigma) (40). After a few days of viral infection, cultural supernatants were collected following the appearance of a significant cytopathic effect (CPE) throughout the monolayer cells, and viral purification was implemented. The prepared viral supernatants were centrifuged at 120,000 × *g* for 20 min to remove cell debris and centrifuged at 140,000 × *g* at 4°C for 120 min to concentrate viruses using a Beckman SW32Ti rotor; the viruses were suspended in HNE buffer (5 mM HEPES, 150 mM NaCl, and 0.1 mM EDTA [pH 7.4]) and purified using sucrose (10% to 60%) gradient ultracentrifugation at 140,000 × *g* for 120 min with the same rotor. Viral bands in sucrose solution were collected and centrifuged to remove sucrose (41). Finally, the purified viruses were resuspended in HNE buffer, aliquoted, and stored at −80°C for later use.

### Virus labeling.

PEDVs were labeled with a lipophilic cationic dye, 1,1′-dioctadecyl-3,3,3′,3′-tetramethylindodicarbocyanine (DiD; Thermo Fisher, USA). A final concentration of 250 μM DiD dye was used to label the purified viruses, and it was first incubated at room temperature for 90 min with a soft vortex to ensure sufficient partition of DiD dye into the viral envelope (23, 24, 42). Next, excess dyes were removed using a NAP-10 filtration column (GE Healthcare). Finally, DiD-labeled PEDVs were filtered through a 0.22-μm pore filter (Millipore), aliquoted, and stored at −80°C.

### Plasmid construction and transfection.

The full-length light chains of clathrin (LCa) (accession number XM_007968752), caveolin 1 (Cav1) (accession number AM_007982675), and epidermal growth factor receptor substrate 15 (Eps15) (accession number XM_007978771) were amplified by PCR using Vero cell cDNA and then inserted into pEGFP-C1, pmKO2-N1, and pEGFP-C3 to generate pLCa-EGFP, pCav1-mKO2, and pEps15-EGFP, respectively. Eps15 homology (EH) domain-deleted pEGFP-Eps15 formed EGFP-tagged Eps15 Δ29/295, which inhibits clathrin-coated vesicle formation. pEGFP-Eps15 was used to construct pEGFP-D3Δ2, which does not perturb CME (43, 44). Cav1-mKO2 was used to construct a caveolin-1 dominant-negative (Cav1-DN) mutant, and the cDNA of Mus musculus was used to construct a caveolin-3 dominant-negative (Cav3-DN) mutant to inhibit CavME (45, 46). Wild-type and dominant-negative Rab5 and Rab7 were generated by PCR from cDNA libraries of 293T cells and then inserted into pEGFP-C1 and pmCherry-C1 to generate pEGFP-Rab5, pmCherry-Rab5 S34N, pmCherry-Rab5 Q79L, pEGFP-Rab7, pmCherry-Rab7 T22N, and pmCherry-Rab7 Q67L plasmids (47). The primers used to construct the plasmids are shown in Table S1 in the supplemental material.

10.1128/mBio.00256-21.9 TABLE S1 Primers used for cloning. Download Table S1, DOCX file, 0.01 MB. Copyright © 2021 Li et al. 2021 Li et al. https://creativecommons.org/licenses/by/4.0/ This content is distributed under the terms of the Creative Commons Attribution 4.0 International license.

### Immunofluorescence assay.

Vero cells were first grown on a 15-mm glass-bottomed cell culture dish (Nest Biotechnology, China) and then transfected with pEGFP-LCa or pmKO2-Cav1. After 24 h, the cells were fixed with 4% paraformaldehyde for 15 min at room temperature, washed three times with phosphate-buffered saline (PBS), permeabilized with 0.3% Triton X-100 for 5 min, and blocked with QuickBlock blocking buffer (Beyotime Biotechnology, China) for 15 min. Next, to label the clathrin-coated structures and caveolae, cells were incubated with rabbit anti-clathrin light-chain IgG primary antibody (1:300 dilutions; Proteintech, China) and rabbit anti-caveolin-1 IgG primary antibody (1:200 dilutions, Proteintech), respectively, for 2 h at room temperature. After being washed three times with PBS, the cells were incubated with Alexa Fluor 546-conjugated goat anti-rabbit IgG secondary antibody (1:500 dilutions; Thermo Fisher, USA) to label clathrin and Alexa Fluor 488-conjugated goat anti-rabbit IgG secondary antibody (1:500 dilutions, Thermo Fisher) to label caveolin 1, respectively. Finally, Hoechst 33342 (Thermo Fisher) was used to stain cellular nuclei.

To visualize DiD-labeled PEDVs using an immunofluorescence assay, 25 μl of viral supernatant was fixed on a glass-bottomed cell culture dish containing Polybrene (8 μg/ml final concentration, using stock 8 mg/ml at 1:1,000; Sigma, USA) to promote the adhesion of virus particles to glass surfaces (48). Then, the mouse anti-PEDV N IgG primary antibody (1:200 dilutions, prepared in our laboratory) and FITC-conjugated goat anti-mouse IgG secondary antibody (1:500 dilutions; KPL, SeraCare, USA) were used to label viruses. Finally, fluorescence images were recorded using a Nikon A1 confocal microscope (Japan).

### Single-virus tracking.

Vero cells were first seeded on a glass-bottomed cell culture dish to reach a 70% to 80% confluence. Then, plasmids were transfected into the cells using LipoMax (Sudgen, China). After transfection for 24 h, the cell culture medium was refreshed with phenol red-free DMEM (Procell Life, China) containing 0.3% TPB, 3 μg/ml trypsin, and 1% ProLong live antifade reagent (Thermo Fisher, USA) before live-cell imaging. Next, in single-virus tracking, DiD-labeled PEDVs infected the cells with a multiplicity of infection (MOI) of 50 and were incubated at 4°C for 30 min to synchronize viral infection (49). The fluorescence signals were collected using a micro-objective (Plan Apo 60×/1.40, oil immersion; Nikon, Japan) in a Nikon A1 plus confocal microscope (Japan), equipped with a stage-top incubator (Tokai Hit, Japan) for a stable cell imaging environment with 5% CO_2_ at 37°C. EGFP, mCherry/mKO2, and DiD were excited using lasers with wavelengths of 488 nm (Melles Griot, USA), 561 nm (Coherent, USA), and 640 nm (Coherent), respectively; their corresponding emission fluorescence signals were collected through bandpass filters with central wavelengths/full width at half maximum of 527/55 nm, 615/70 nm, and 707/90 nm, respectively. The laser powers were low, only a few milliwatts, to guarantee long imaging periods. Live-cell TIRF imaging was performed using a commercial TIRF microscope (Nikon) equipped with a Plan Apo TIRF 100×/1.49 NA oil immersion micro-objective (Nikon). The excitation lasers, emission filters, and cell imaging environment were the same as for confocal imaging. Fluorescence signals were collected using an EMCCD camera (iXon3, Andor). Finally, in imaging analysis, time-lapse images were deconvoluted with a Gaussian filter. Kymographs and time traces of fluorescence intensities were obtained from these time-lapse series using NIS-Elements AR 4.51.00 (Nikon). Trajectories and velocities of viruses were analyzed using the MTrackJ plugin in Fiji/ImageJ; MSD was calculated using an in-house Matlab program (MathWorks, USA), and movement modes were determined by binomially fitting of MSD time plots (50).

### Transmission electron microscopy.

To visualize the cellular morphology in viral infection, cells were first incubated with PEDVs at a high MOI of 1,000 for 30 min at 4°C, and the cultures were then transferred to 37°C. After infection with PEDV for 30 min at 37°C, cells were next fixed with 2.5% glutaraldehyde for 1 h at room temperature; finally, cells were collected. Next, the specimens were processed for ultrathin-section preparation (51). Finally, these ultrathin (70 to 80 nm) sections of cells were observed using TEM (Tecnai G 2 Spirit BioTWIN; FEI, USA).

### Double-label immunoelectron microscopy.

Cells were first incubated with PEDVs at a high MOI of 1,000 for 30 min at 4°C, then transferred to 37°C, fixed with immune electron microscopy fixative (G1124; Servicebio) for 2 h at room temperature, and finally collected. Double-label immunoelectron microscopy was performed with standard procedures. Briefly, the collected cell precipitation was resuspended and washed twice with precooled 0.1 M PBS (pH 7.4). After the supernatant was removed, samples were processed with dehydrating, resin penetrating, embedding and polymerizing steps. Sample resin blocks were sliced into 90-nm ultrathin cryosections using a Leica FC7 ultramicrotome and collected onto the 150-mesh nickel grids with Formvar film for immunogold labeling. The nickel grids were incubated with 1:100 dilution of rabbit anti-clathrin light chain antibody (ab150658; Abcam) and 1:50 dilution of mouse anti-caveolin-1 antibody (ab17052; Abcam) overnight at 4°C. The nickel grids were rinsed with PBS 3 times at 5 min each and then were incubated with a 1:50 dilution of gold-conjugated goat anti-rabbit IgG (G7402, 10 nm; Sigma) and 1:100 dilution of gold-conjugated goat anti-mouse IgG (115-185-146, 4 nm; Jackson) for 1 h at 37°C. The grids were washed and stained with 2% uranyl acetate. Finally, the sections were examined with a transmission electron microscope (HT7800; Hitachi).

### Internalization assay for transferrin, CTB, and PEDV entry.

For the internalization assay, Vero cells were first seeded onto a glass-bottomed cell culture dish and transfected with wild-type and/or dominant-negative plasmids. After 24 h, cells were incubated with Alexa 546-conjugated transferrin (10 μg/ml; Thermo Fisher, USA), Alexa 647-conjugated CTB (10 μg/ml; Thermo Fisher), and DiD-labeled PEDVs (MOI 10) at 4°C for 20 min. Then, the specimens were kept at 37°C for 30 min. Next, internalization was blocked by ice-cold DMEM washes. From the cell membrane, noninternalized endocytic markers and viruses were removed using 0.1 M glycine and a 0.1 M NaCl solution (pH 3.0). Finally, cells were fixed with 4% paraformaldehyde for 15 min at room temperature and observed with a Nikon A1 plus confocal microscope (Japan). Fluorescence images were recorded from at least three separate samples. The average fluorescence intensity was quantified using NIS-Elements AR 4.51.00 (Nikon).

### Western blot.

Cells were washed twice with PBS (pH 7.2) and lysed with NP-40 lysis buffer containing 1 mM protease inhibitor phenylmethanesulfonyl fluoride (Beyotime, China). After samples were incubated on ice for 15 min, the cell lysates were collected by centrifugation at 10,000 × *g* for 10 min, and the protein content was measured using bicinchoninic acid (BCA) protein assay kits (GenStar, China). Then, equalized amounts of proteins were separated by sodium dodecyl sulfate-polyacrylamide gel electrophoresis (SDS-PAGE), transferred to 0.2-μm nitrocellulose blot membranes (GE Healthcare, UK), and blocked using 5% nonfat milk for 1 h at room temperature. Next, the membranes were incubated with various primary antibodies and horseradish peroxidase (HRP)-conjugated secondary antibodies. Finally, the membranes were detected using the enhanced chemiluminescence (ECL) buffer (Vazyme, China).

## References

[1] Cui J, Li F, Shi Z-L. 2019. Origin and evolution of pathogenic coronaviruses. Nat Rev Microbiol 17:181–192. doi:10.1038/s41579-018-0118-9. 30531947 PMC7097006

[2] Docea AO, Tsatsakis A, Albulescu D, Cristea O, Zlatian O, Vinceti M, Moschos SA, Tsoukalas D, Goumenou M, Drakoulis N, Dumanov JM, Tutelyan VA, Onischenko GG, Aschner M, Spandidos DA, Calina D. 2020. A new threat from an old enemy: re-emergence of coronavirus. Int J Mol Med 45:1631–1643. doi:10.3892/ijmm.2020.4555. 32236624 PMC7169834

[3] Wang L, Wang Y, Ye D, Liu Q. 2020. Review of the 2019 novel coronavirus (SARS-CoV-2) based on current evidence. Int J Antimicrob Agents 55:105948. doi:10.1016/j.ijantimicag.2020.105948. 32201353 PMC7156162

[4] Andersen KG, Rambaut A, Lipkin WI, Holmes EC, Garry RF. 2020. The proximal origin of SARS-CoV-2. Nat Med 26:450–452. doi:10.1038/s41591-020-0820-9. 32284615 PMC7095063

[5] Tang X, Wu C, Li X, Song Y, Yao X, Wu X, Duan Y, Zhang H, Wang Y, Qian Z, Cui J, Lu J. 2020. On the origin and continuing evolution of SARS-CoV-2. Natl Sci Rev 7:1012–1023. doi:10.1093/nsr/nwaa036. PMC7107875 34676127

[6] Lee C. 2015. Porcine epidemic diarrhea virus: an emerging and re-emerging epizootic swine virus. Virol J 12:193. doi:10.1186/s12985-015-0421-2. 26689811 PMC4687282

[7] Song D, Moon H, Kang B. 2015. Porcine epidemic diarrhea: a review of current epidemiology and available vaccines. Clin Exp Vaccine Res 4:166–176. doi:10.7774/cevr.2015.4.2.166. 26273575 PMC4524901

[8] Jung K, Saif LJ. 2015. Porcine epidemic diarrhea virus infection: etiology, epidemiology, pathogenesis and immunoprophylaxis. Vet J 204:134–143. doi:10.1016/j.tvjl.2015.02.017. 25841898 PMC7110711

[9] Hou Y, Wang Q. 2019. Emerging highly virulent porcine epidemic diarrhea virus: molecular mechanisms of attenuation and rational design of live attenuated vaccines. Int J Mol Sci 20:5478. doi:10.3390/ijms20215478. PMC6862049 31689903

[10] Li S, Yang J, Zhu Z, Zheng H. 2020. Porcine epidemic diarrhea virus and the host innate immune response. Pathogens 9:367. doi:10.3390/pathogens9050367. PMC7281546 32403318

[11] Chen F, Pan Y, Zhang X, Tian X, Wang D, Zhou Q, Song Y, Bi Y. 2012. Complete genome sequence of a variant porcine epidemic diarrhea virus strain isolated in China. J Virol 86:12448. doi:10.1128/JVI.02228-12. 23087112 PMC3486467

[12] Li W, van Kuppeveld FJM, He Q, Rottier PJM, Bosch B-J. 2016. Cellular entry of the porcine epidemic diarrhea virus. Virus Res 226:117–127. doi:10.1016/j.virusres.2016.05.031. 27317167 PMC7114534

[13] Park J-E, Cruz DJM, Shin H-J. 2014. Clathrin- and serine proteases-dependent uptake of porcine epidemic diarrhea virus into Vero cells. Virus Res 191:21–29. doi:10.1016/j.virusres.2014.07.022. 25086180 PMC7114442

[14] Jeon JH, Lee C. 2017. Cellular cholesterol is required for porcine nidovirus infection. Arch Virol 162:3753–3767. doi:10.1007/s00705-017-3545-4. 28884395 PMC7086867

[15] Wei X, She G, Wu T, Xue C, Cao Y. 2020. PEDV enters cells through clathrin-, caveolae-, and lipid raft-mediated endocytosis and traffics via the endo-/lysosome pathway. Vet Res 51:10. doi:10.1186/s13567-020-0739-7. 32041637 PMC7011528

[16] Mettlen M, Chen P-H, Srinivasan S, Danuser G, Schmid SL. 2018. Regulation of clathrin-mediated endocytosis. Annu Rev Biochem 87:871–896. doi:10.1146/annurev-biochem-062917-012644. 29661000 PMC6383209

[17] Kaksonen M, Roux A. 2018. Mechanisms of clathrin-mediated endocytosis. Nat Rev Mol Cell Biol 19:313–326. doi:10.1038/nrm.2017.132. 29410531

[18] Cheng JPX, Nichols BJ. 2016. Caveolae: one function or many? Trends Cell Biol 26:177–189. doi:10.1016/j.tcb.2015.10.010. 26653791

[19] Parton RG. 2018. Caveolae: structure, function, and relationship to disease. Annu Rev Cell Dev Biol 34:111–136. doi:10.1146/annurev-cellbio-100617-062737. 30296391

[20] Brandenburg B, Zhuang X. 2007. Virus trafficking—learning from single-virus tracking. Nat Rev Microbiol 5:197–208. doi:10.1038/nrmicro1615. 17304249 PMC2740720

[21] Liu S-L, Wang Z-G, Xie H-Y, Liu A-A, Lamb DC, Pang D-W. 2020. Single-virus tracking: from imaging methodologies to virological applications. Chem Rev 120:1936–1979. doi:10.1021/acs.chemrev.9b00692. 31951121 PMC7075663

[22] Mattheyses AL, Simon SM, Rappoport JZ. 2010. Imaging with total internal reflection fluorescence microscopy for the cell biologist. J Cell Sci 123:3621–3628. doi:10.1242/jcs.056218. 20971701 PMC2964103

[23] Lakadamyali M, Rust MJ, Babcock HP, Zhuang X. 2003. Visualizing infection of individual influenza viruses. Proc Natl Acad Sci U S A 100:9280–9285. doi:10.1073/pnas.0832269100. 12883000 PMC170909

[24] Spence JS, Krause TB, Mittler E, Jangra RK, Chandran K. 2016. Direct visualization of ebola virus fusion triggering in the endocytic pathway. mBio 7:e01857-15. doi:10.1128/mBio.01857-15. 26861015 PMC4752599

[25] Hu Y-B, Dammer EB, Ren R-J, Wang G. 2015. The endosomal-lysosomal system: from acidification and cargo sorting to neurodegeneration. Transl Neurodegener 4:18. doi:10.1186/s40035-015-0041-1. 26448863 PMC4596472

[26] White JM, Whittaker GR. 2016. Fusion of enveloped viruses in endosomes. Traffic 17:593–614. doi:10.1111/tra.12389. 26935856 PMC4866878

[27] Schelhaas M. 2010. Come in and take your coat off—how host cells provide endocytosis for virus entry. Cell Microbiol 12:1378–1388. doi:10.1111/j.1462-5822.2010.01510.x. 20678171

[28] Helenius A. 2018. Virus entry: looking back and moving forward. J Mol Biol 430:1853–1862. doi:10.1016/j.jmb.2018.03.034. 29709571 PMC7094621

[29] Inoue Y, Tanaka N, Tanaka Y, Inoue S, Morita K, Zhuang M, Hattori T, Sugamura K. 2007. Clathrin-dependent entry of severe acute respiratory syndrome coronavirus into target cells expressing ACE2 with the cytoplasmic tail deleted. J Virol 81:8722–8729. doi:10.1128/JVI.00253-07. 17522231 PMC1951348

[30] Pu Y, Zhang X. 2008. Mouse hepatitis virus type 2 enters cells through a clathrin-mediated endocytic pathway independent of Eps15. J Virol 82:8112–8123. doi:10.1128/JVI.00837-08. 18550663 PMC2519582

[31] Owczarek K, Szczepanski A, Milewska A, Baster Z, Rajfur Z, Sarna M, Pyrc K. 2018. Early events during human coronavirus OC43 entry to the cell. Sci Rep 8:7124. doi:10.1038/s41598-018-25640-0. 29740099 PMC5940804

[32] Wang H, Yuan X, Sun Y, Mao X, Meng C, Tan L, Song C, Qiu X, Ding C, Liao Y. 2019. Infectious bronchitis virus entry mainly depends on clathrin mediated endocytosis and requires classical endosomal/lysosomal system. Virology 528:118–136. doi:10.1016/j.virol.2018.12.012. 30597347 PMC7111473

[33] Laniosz V, Holthusen KA, Meneses PI. 2008. Bovine papillomavirus type 1: from clathrin to caveolin. J Virol 82:6288–6298. doi:10.1128/JVI.00569-08. 18417596 PMC2447075

[34] He K, Yan X, Li N, Dang S, Xu L, Zhao B, Li Z, Lv Z, Fang X, Zhang Y, Chen Y-G. 2015. Internalization of the TGF-β type I receptor into caveolin-1 and EEA1 double-positive early endosomes. Cell Res 25:738–752. doi:10.1038/cr.2015.60. 25998683 PMC4456627

[35] Sun E-Z, Liu A-A, Zhang Z-L, Liu S-L, Tian Z-Q, Pang D-W. 2017. Real-time dissection of distinct dynamin-dependent endocytic routes of influenza A virus by quantum dot-based single-virus tracking. ACS Nano 11:4395–4406. doi:10.1021/acsnano.6b07853. 28355058

[36] Jovic M, Sharma M, Rahajeng J, Caplan S. 2010. The early endosome: a busy sorting station for proteins at the crossroads. Histol Histopathol 25:99–112. doi:10.14670/HH-25.99. 19924646 PMC2810677

[37] Vonderheit A, Helenius A. 2005. Rab7 associates with early endosomes to mediate sorting and transport of Semliki Forest virus to late endosomes. PLoS Biol 3:e233. doi:10.1371/journal.pbio.0030233. 15954801 PMC1151600

[38] Hoornweg TE, van Duijl-Richter MKS, Ayala Nuñez NV, Albulescu IC, van Hemert MJ, Smit JM. 2016. Dynamics of Chikungunya virus cell entry unraveled by single-virus tracking in living cells. J Virol 90:4745–4756. doi:10.1128/JVI.03184-15. 26912616 PMC4836339

[39] Liu J, Xu M, Tang B, Hu L, Deng F, Wang H, Pang D-W, Hu Z, Wang M, Zhou Y. 2019. Single-particle tracking reveals the sequential entry process of the bunyavirus severe fever with thrombocytopenia syndrome virus. Small 15:1803788. doi:10.1002/smll.201803788. 30589216

[40] Hofmann M, Wyler R. 1988. Propagation of the virus of porcine epidemic diarrhea in cell culture. J Clin Microbiol 26:2235–2239. doi:10.1128/JCM.26.11.2235-2239.1988. 2853174 PMC266866

[41] Leibowitz J, Kaufman G, Liu P. 2011. Coronaviruses: propagation, quantification, storage, and construction of recombinant mouse hepatitis virus. Curr Protoc Microbiol Chapter 15:Unit 15E.1. 10.1002/9780471729259.mc15e01s21 PMC3119930 21538303

[42] He J, Sun E, Bujny MV, Kim D, Davidson MW, Zhuang X. 2013. Dual function of CD81 in influenza virus uncoating and budding. PLoS Pathog 9:e1003701. doi:10.1371/journal.ppat.1003701. 24130495 PMC3795033

[43] Benmerah A, Poupon V, Cerf-Bensussan N, Dautry-Varsat A. 2000. Mapping of Eps15 domains involved in its targeting to clathrin-coated pits. J Biol Chem 275:3288–3295. doi:10.1074/jbc.275.5.3288. 10652316

[44] Benmerah A, Bayrou M, Cerf-Bensussan N, Dautry-Varsat A. 1999. Inhibition of clathrin-coated pit assembly by an Eps15 mutant. J Cell Sci 112:1303. 10194409 10.1242/jcs.112.9.1303

[45] Trouet D, Hermans D, Droogmans G, Nilius B, Eggermont J. 2001. Inhibition of volume-regulated anion channels by dominant-negative caveolin-1. Biochem Biophys Res Commun 284:461–465. doi:10.1006/bbrc.2001.4995. 11394902

[46] Roy S, Luetterforst R, Harding A, Apolloni A, Etheridge M, Stang E, Rolls B, Hancock JF, Parton RG. 1999. Dominant-negative caveolin inhibits H-Ras function by disrupting cholesterol-rich plasma membrane domains. Nat Cell Biol 1:98–105. doi:10.1038/10067. 10559881

[47] Krzyzaniak MA, Zumstein MT, Gerez JA, Picotti P, Helenius A. 2013. Host cell entry of respiratory syncytial virus involves macropinocytosis followed by proteolytic activation of the F protein. PLoS Pathog 9:e1003309. doi:10.1371/journal.ppat.1003309. 23593008 PMC3623752

[48] Pizzato M, Marlow SA, Blair ED, Takeuchi Y. 1999. Initial binding of murine leukemia virus particles to cells does not require specific Env-receptor interaction. J Virol 73:8599–8611. doi:10.1128/JVI.73.10.8599-8611.1999. 10482613 PMC112880

[49] Padilla-Parra S, Marin M, Kondo N, Melikyan GB. 2014. Pinpointing retrovirus entry sites in cells expressing alternatively spliced receptor isoforms by single virus imaging. Retrovirology 11:47. doi:10.1186/1742-4690-11-47. 24935247 PMC4065388

[50] Wang J, Li Y, Wang S, Liu F. 2020. Dynamics of transmissible gastroenteritis virus internalization unraveled by single-virus tracking in live cells. FASEB J 34:4653–4669. doi:10.1096/fj.201902455R. 32017270 PMC7163995

[51] Oka T, Saif LJ, Marthaler D, Esseili MA, Meulia T, Lin C-M, Vlasova AN, Jung K, Zhang Y, Wang Q. 2014. Cell culture isolation and sequence analysis of genetically diverse US porcine epidemic diarrhea virus strains including a novel strain with a large deletion in the spike gene. Vet Microbiol 173:258–269. doi:10.1016/j.vetmic.2014.08.012. 25217400 PMC7126216

