# Supplementary figures and images for: Dynamic Dissection of the Endocytosis of Porcine Epidemic Diarrhea Coronavirus Cooperatively Mediated by Clathrin and Caveolae as Visualized by Single-Virus Tracking
Source: mBio. 2021 Mar 30;12(2):e00256-21. doi: 10.1128/mBio.00256-21 (PMC8092227; doi:10.1128/mBio.00256-21)

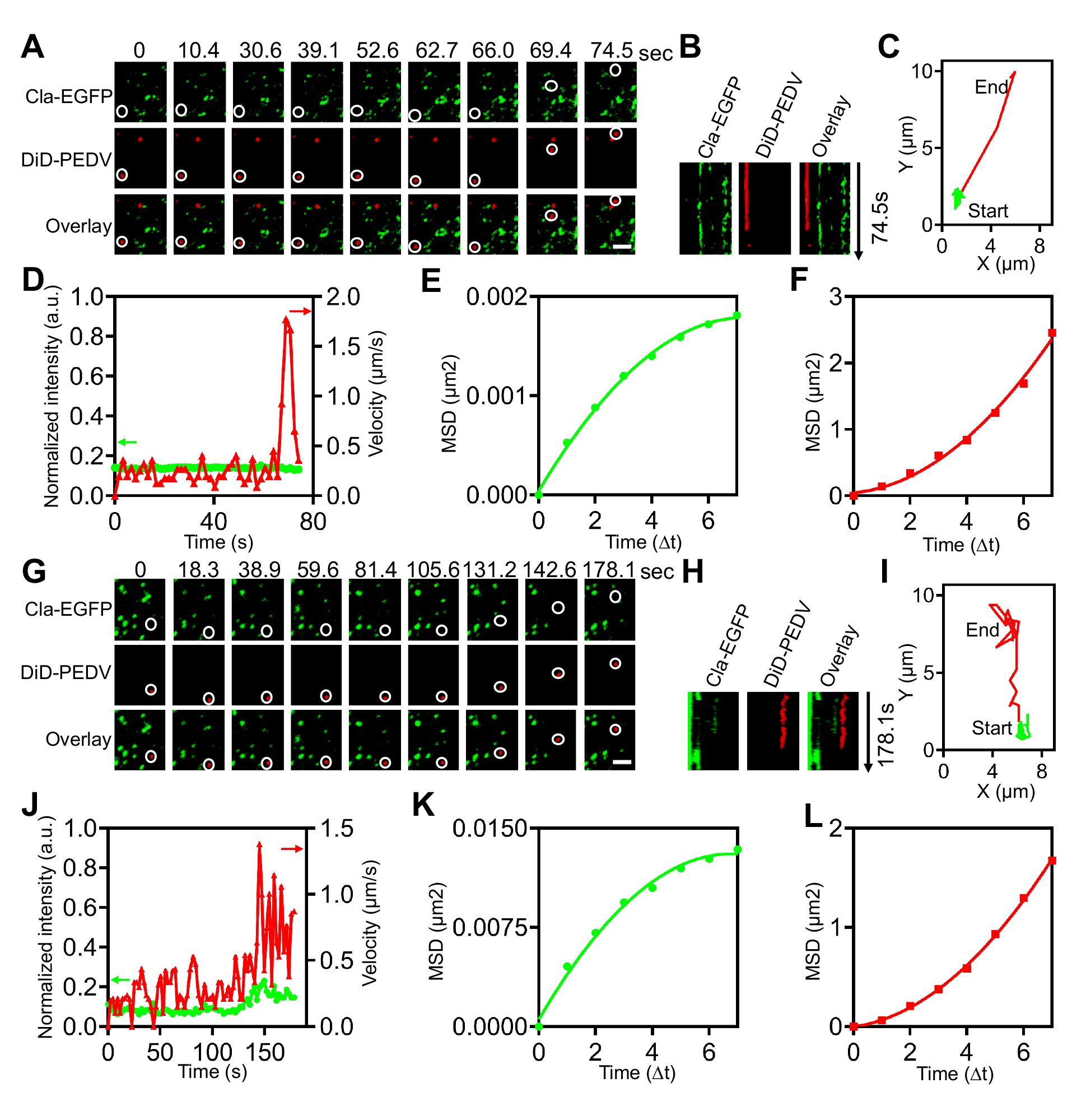

Supplement: FIG S1 [file mBio.00256-21-sf001.tif]

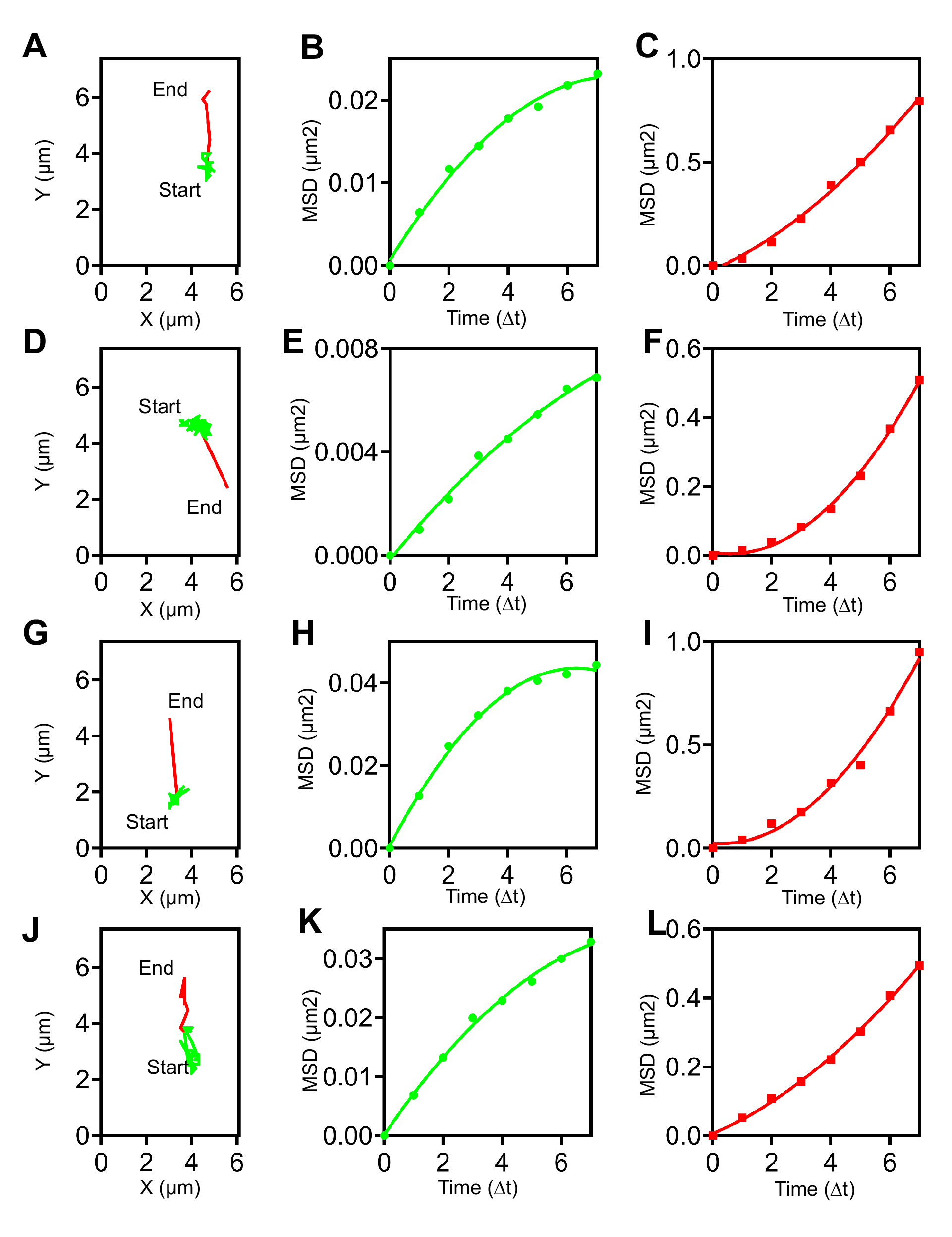

Supplement: FIG S2 [file mBio.00256-21-sf002.tif]
