# Supplementary material for: Dynamic Dissection of the Endocytosis of Porcine Epidemic Diarrhea Coronavirus Cooperatively Mediated by Clathrin and Caveolae as Visualized by Single-Virus Tracking
Source: mBio. 2021 Mar 30;12(2):e00256-21. doi: 10.1128/mBio.00256-21 (PMC8092227; doi:10.1128/mBio.00256-21)
Supplement: TABLE S1 [file mBio.00256-21-st001.docx]

**Table S1.** Primers used for cloning.

| Name | Primer sequence (5′-3′) | Vector |
| --- | --- | --- |
| Cla | F: GGAATTCTGCTGAGCTGGATCCATTCGGC  R: GGTACCTCAGTGCACCAGCGGGGCCT | pEGFP-C1 |
| Eps 15 | F: CGCCTCGAGATGGCTGCGGCGGCCCAG  R: CGCGGTACCGTTCATGCTTCTGATATCTCA  GATTTG | pEGFP-C3 |
| Eps15 DN | F: CCACCAAGATTTCATGATACCAGTATAATC  AGTCAGAAGTTAA  R: CCCTTGATTAACTTCTGACTGATTATACTGGT  ATCATGAAATC | pEGFP-C3 |
| DIIIΔ2 | F: TGCAGATACAAACTTGGATTTTTTCAGTTCAG  CCACATCGAGC  R: TTACAGAGCTCGATGTGGCTGAACTGAAAAAAT  CCAAGTTTGT | pEGFP-C3 |
| Cav1 | F: GCTCGAGATGCTCCCTTGTCGCGG  R: GGAATTCGTATTTCTTTCTGCAAGTTGATG | pmKO2-N1 |
| Cav1-DN | F: GCTCGAGATGGTGGTCAAGATTGACTTTG  R: GGAATTCGTATTTCTTTCTGCAAGTTGATG | pmKO2-N1 |
| Cav3-DN | F: GCTCGAGATGGACGGTGTATGGAAGGTGAG  R: GGAATTCGGCCTTCCCTTCGCAGCACCACC | pEGFP-N1 |
| Rab5 | F: GGGTACCATGGCTAGTCGAGGCGCAAC  R: GGGATCCTTAGTTACTACAACACTGATTCCTG | pEGFP-C1 /pmCherry-C1 |
| Rab5-DN(S34N) | F: TGGCAAAAACAGCCTAGTGCTTCGTTTTGTGAA  R: CTAGGCTGTTTTTGCCAACAGCGGACTCTC | pmCherry-C1 |
| Rab5-CA(Q79L) | F: AGCTGGTCTCGAACGATACCATAGCCTAGCACC  R: TATCGTTCGAGACCAGCTGTATCCCATATTTCAA | pmCherry-C1 |
| Rab7 | F: GGGTACCATGACCTCTAGGAAGAAAGTGTTGC  R: GGGATCCTCAGCAACTGCAGCTTTCTGC | pEGFP-C1 /pmCherry-C1 |
| Rab7-DN(T22N) | F: CGGGAAGAACTCACTCATGAACCAGTATGTGA  ATAAGA  R: ATGAGTGAGTTCTTCCCGACTCCAGAATCTCC | pmCherry-C1 |
| Rab7-CA(Q67L) | F: ACACAGCAGGACTGGAACGGTTCCAGTCTCT  CGG  R: TTCCAGTCCTGCTGTGTCCCATATCTGC | pmCherry-C1 |
